# Distal amino-acid substitutions contribute to HIV protease inhibitor resistance as directly as proximal amino-acid substitutions

**DOI:** 10.1038/s41598-026-64620-7

**Published:** 2026-10-05

**Authors:** Debananda Das, Hironori Hayashi, Yuki Takamatsu, Manabu Aoki, Haydar Bulut, Ravikiran S. Yedidi, Kazutaka Murayama, Kazuya Hasegawa, Arun K. Ghosh, Hiroaki Mitsuya

**Affiliations:** 1 Experimental Retrovirology Section, HIV and AIDS Malignancy Branch, National Cancer Institute, National Institutes of Health, Bethesda, MD 20892 USA; 2 Department of Intelligent Network for Infection Control, Tohoku University Graduate School of Medicine, Miyagi, Japan; 3 Department of Infectious Diseases, International Research Institute of Disaster Science, Tohoku University, Miyagi, Japan; 4 Department of Refractory Viral Diseases, Japan Institute for Health Security, Tokyo, 162-8655 Japan; 5 Department of Medical Technology, Kumamoto Health Science University, Kumamoto, 861-5598 Japan; 6 Division of Biomedical Measurements and Diagnostics, Graduate School of Biomedical Engineering, Tohoku University, Sendai, Japan; 7 Diffraction and Scattering Division, Japan Synchrotron Radiation Research Institute, Hyogo, Japan; 8 Departments of Chemistry and Medicinal Chemistry, Purdue University, West Lafayette, IN 47907 USA; 9 Departments of Infectious Diseases and Hematology, Kumamoto University Hospital, Kumamoto, 860-8556 Japan; 10 Present Address: TCABS-E Laboratories, Visakhapatnam, AP 530003 India

**Keywords:** HIV resistance, Protease inhibitor resistance mechanism, Darunavir, GRL142, Molecular dynamics, Distal amino acid substitutions, Biochemistry, Drug discovery, Microbiology, Structural biology

## Abstract

There are two categories of drug-resistance-associated amino-acid substitutions responsible for causing drug resistance in HIV-1 protease: (i) major and (ii) minor substitutions. In general, major substitutions mostly arise early during treatment, reduce susceptibility to the drug and are mostly proximal to the protease active site. Minor substitutions mostly arise later, may provide incremental resistance and/or improve viral fitness and are often distal from the active site. However, no precise definitions of such two categories exist. Herein, using X-ray crystallography and molecular dynamics (MD) simulations we examined the structural basis of antiviral resistance of HIV_DRV_^R^_p51_, an HIV-1 variant that has three proximal (V32I, V82I, I84V) and eleven distal substitutions (L10I/I15V/K20R/L24I/L33F/M36I/ M46L/ I54M/L63P/K70Q/L89M) in the protease region (PR_p51_), and has high resistance against a protease inhibitor darunavir (DRV), but not against another protease inhibitor GRL142. MD demonstrated that the proximal and distal substitutions work in concert to carry out structural changes that result in larger perturbations in inter-residue distances and active site cavity volume for DRV-PR_p51_ over DRV-PR_wt_ compared to GRL142-PR_p51_ over GRL142-PR_wt_. These results provided a rationale for the different resistance profiles of DRV and GRL142. The results also suggest reconsideration of the simplistic classification of substitutions as either major or minor.

**Supplementary Information:**

The online version contains supplementary material available at https://doi.org/10.1038/s41598-026-64620-7.

## Introduction

As of July 2025, roughly forty million people are living with HIV infection worldwide, and nearly thirty-one million are on anti-retroviral therapy (ART) (https://www.who.int/news-room/fact-sheets/detail/hiv-aids and https://www.who.int/news-room/fact-sheets/detail/hiv-drug-resistance). Most of ART targets HIV viral proteins such as reverse transcriptase (RT), protease (PR), and integrase (IN) and prevent viral replication. New anti-HIV targets and interventions continue to be explored continually^1,2^. Current research focuses on not only improving the safety and resistance profile of newer anti-HIV inhibitors, but also in targeting viral reservoirs and in developing antivirals that are effective at longer intervals of dosing^3^.

**Fig. 1:**
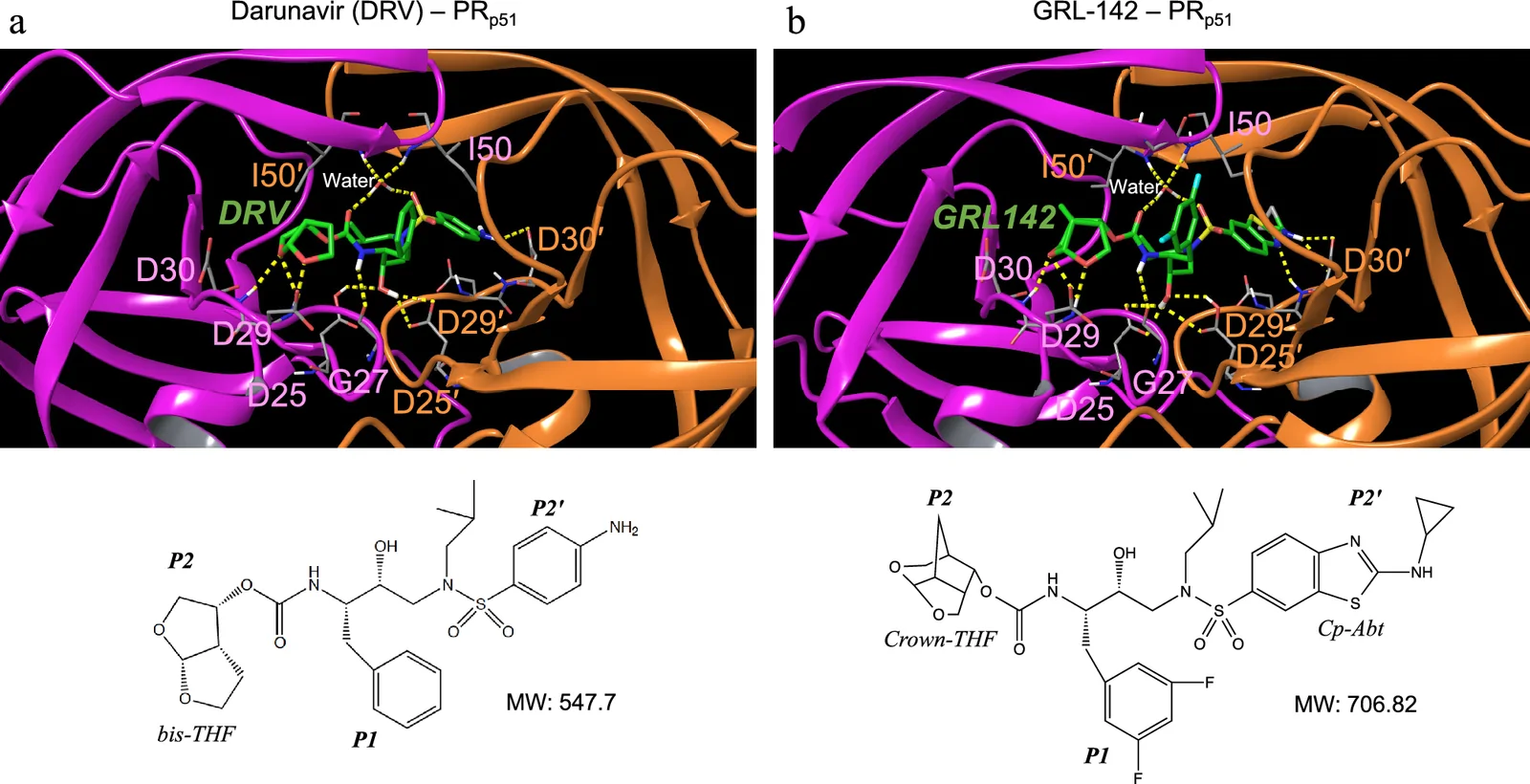
DRV and GRL142 have hydrogen bond interactions with the same residues of PR_p51_. The hydrogen bond interactions of DRV with PR_p51_ (panel a, PDB ID: 9CTX) and of GRL142 with PR_p51_ (panel b, PDB ID:6MKL) from their respective crystal structures are shown. Though DRV and GRL142 have different P2 and P2′ groups, they form hydrogen bond interactions with the same residues of PR_p51_, with GRL142 having an additional hydrogen bond interaction with Asp30′. The protease dimer is shown in purple and orange ribbons. DRV and GRL142 are shown in thick sticks, with green carbons. Protease residues are shown in gray carbons. Hydrogen bonds are shown in yellow dashed lines.

Darunavir (DRV), an HIV-protease inhibitor (PI), has exceedingly high potency against wild-type and drug-resistant HIV^4,5^ (Fig. 1). DRV was approved by the US-FDA for clinical use in 2007, first for use by patients failing other anti-HIV therapies because of drug resistance, and later in drug-naïve patients^6^. DRV is also used as part of combination therapy regimens with RT and IN inhibitors. DRV has a high genetic barrier to HIV-1’s development of resistance. HIV carrying single mutations such as L10F, L24I, V32I, L33F, M46I, I54M, V82A, I84V, with two mutations such as V32I and I84V, or V32I and I54M, and with three mutations as in V32I, L33F, and I84V is still sensitive to DRV^7–9^. Though DRV has a high genetic barrier to HIV-1’s development of resistance, multiple mutations in the protease, especially in treatment-experienced patients, can lead to HIV developing significant resistance^10^. Thus, attempts have been made to identify new protease inhibitors with improved potency, resistance, and safety profile. GRL142 is one such PI that contained a 6–5-5 ring-fused crown-like tetrahydropyranofuran (Crn-THF) as the P2-ligand, a cyclopropyl-amino-benzothiazole (Cp-Abt) as the P2’-ligand, and a bis-fluoro-benzyl group as the P1-ligand^8^ (Fig. 1). GRL142 had subnanomolar activity against HIV_wt_ and many drug-resistant HIV variants^8^. Compared to many other PIs, GRL142 had a higher genetic barrier against the development of resistance by HIV-1, had excellent cytotoxicity profile and high selectivity index^8^. Drug resistant mutations that compromise the activity of GRL142 under a clinical setting have not been reported so far to the best of our knowledge. Surprisingly, HIV-1 with single mutants V32I, I54M, V82A and I84V were more sensitive to GRL142^8^.

A drug-resistant (DR) amino-acid (AA) substitution is typically categorized as a major substitution if it arises early during a treatment and severely compromises the efficacy of the drug. The minor substitutions tend to accrue after major substitutions^10^. Minor substitutions may add incremental resistance and/or improve the replication fitness of the virus. For the nine protease inhibitors approved by the United States Food and Drug Administration for clinical use, HIV-1 developed resistance by a combination of major and minor mutations in the protease^10^. Mutations V82A and I84V caused resistance against atazanavir (ATZ), lopinavir (LPV) and indinavir (IDV); mutation of Leu90 to a methionine developed resistance against nelfinavir (NFV) and saquinavir (SQV)^10^. V82A and I84V are classified as major resistant mutations for ATZ, LPV and IDV^10^. L90M is classified as a major mutation for NFV and SQV^10^. Though L10F was reported as a minor drug-resistant mutation for ATZ, LPV, and NFV, it has not been reported as a resistant mutation for patients receiving DRV^10^. T74P was reported as a major resistant mutation for tipranavir (TPV), but as a minor mutation for DRV, but not reported as a resistant mutation for other PIs^10^. L89V was reported as a minor mutation for both DRV and TPV but not reported for other PIs^10^.

Almost all the major AA substitutions for HIV-1 protease are active site residues that have a change in their polar and/or non-polar binding interactions with the inhibitor. Mostly, the amino acid residues classified as minor substitutions are distal from the active site. Hence, they do not have direct polar or non-polar interactions with the inhibitor and how they structurally impact binding and cause resistance is not clear. At least one study suggested that both major as well as the so called minor substitutions contributed to resistance, and in some cases the minor substitutions, as a group, may contribute more to resistance than major substitutions^11^. Over the years, there is an increasing realization that the current classification of major and minor substitutions is not clear cut and might be oversimplified^10,11^.

HIV-1 was not able to select resistant variants against DRV when resistant selection experiments were carried out using either HIV_wt_ or with single HIV-1 isolates^12^. However, resistance selection experiments carried out with a mixture of eight HIV-1 variants that had multiple amino acid mutations that made them resistant to other PIs, but not initially to DRV, ultimately resulted in a variant that was extremely resistant to DRV after 51 weeks^12^. This HIV-1 variant was called HIV_DRV_^R^_p51_. The protease region of HIV_DRV_^R^_p51_, henceforth referred as PR_p51_, had fourteen amino acid substitutions. These substitutions were Leu10Ile, Ile15Val, Lys20Arg, Leu24Ile, Val32Ile, Leu33Phe, Met36Ile, Met46Leu, Ile54Met, Leu63Pro, Lys70Gln, Val82Ile, Ile84Val and Leu89Met. Of these substitutions, three (Val32Ile, Val82Ile and Ile84Val) were proximal to the DRV binding site, the other eleven may be considered distal from the DRV binding site.

In the current study, we used enzyme inhibition assays, antiviral assays, x-ray crystallography and molecular dynamics (MD) simulations for 1500 nano seconds to examine the structural basis of the resistance of HIV_DRV_^R^_p51_ and PR_p51_ against DRV and GRL142. Our data strongly suggested that the combination of major and minor substitutions resulted in larger structural perturbations and larger expansion of the active site volume of DRV-PR_p51_ over DRV-PR_wt_ compared to the structural perturbations and active site volume expansion of GRL142-PR_p51_ over GRL142-PR_wt_. The data also helped explain the different resistance profiles of PR_p51_ against DRV and GRL142.

## Results

**PR**_**p51**_** and HIV**_**p51**_** are highly resistant to darunavir.** HIV_DRV_^R^_p51_ (abbreviated henceforth as HIV_p51_) had fourteen amino acid substitutions in its protease (PR_p51_)^12^ region. We first determined the inhibition of PR_p51_ by DRV and by GRL142. As shown in Table 1, the K_i_ values of DRV and GRL142 against PR_p51_ were 18.7 nM and 0.604 nM, respectively. The activities of DRV and GRL142 against the wild-type protease enzyme (PR_wt_) had been determined previously (Table 1)^13^. Comparison of the K_i_ values indicated that DRV and GRL142 had 1,432- and 43-fold reduction in activity against PR_p51_ over PR_wt_. We also determined the antiviral activities of these inhibitors against the wild-type HIV (HIV_wt_) and HIV_p51_. Compared to its antiviral EC_50_ of 3.2 nM against HIV_wt_, DRV had an EC_50_ value of 1,130 nM against HIV_p51_, a significant reduction in potency by 353-fold. The antiviral EC_50_ values of GRL142 against HIV_wt_ and HIV_p51_ were 0.016 nM and 1.4 nM, respectively, indicating a reduction in GRL142’s potency was only 88-fold. In summary, DRV had significant reductions in enzymatic and antiviral activities against PR_p51_ and HIV_p51_, respectively. In contrast, GRL142 maintained good potency and had much smaller fold reductions in its K_i_ and EC_50_ values for PR_p51_ and HIV_p51_, respectively, compared to its potency against the corresponding wild-type species.

**Table 1:** Enzymatic and antiviral activities of DRV and GRL142 against PR_wt_, PR_p51_, HIV_wt_ and HIV_p51_.

Compound	K_i_ (nM) (PR_wt_)^a^	K_i_ (nM) (PR_p51_)	K_i_ fold change	EC_50_^*b*^, (nM) (HIV_wt_)	EC_50_^b^, (nM) (HIV _p51_^*c*^)	EC_50_ fold change
DRV	0.013	18.7	1,438	3.2 ± 0.1	1,130 ± 292	353
GRL142	0.014	0.604	43.1	0.016 ± 0.017	1.4 ± 0.6	88

^a^Reference^11^.

^*b*^Data shown represent mean EC_50_ values (± 1 standard deviation) derived from the results of three independent experiments conducted in triplicate.

^*c*^The amino acid substitutions identified in protease of HIV_P51_ were as follows: L10I/I15V/K20R/L24I/V32I/L33F/M36I/M46L/I54M/L63P/K70Q/V82I/I84V/L89M.

**Polar and non-polar interactions of DRV and GRL142 with PR**_**p51**_** were similar to their respective interactions with wild-type protease (PR**_**wt**_**).** In the current study, we determined the X-ray crystal structures of PR_p51_ in complex with DRV (PDB ID: 9CTX), and with GRL142 (PDB ID: 6MKL). The crystal structure parameters are given in supplementary Table 1. The crystal structures of DRV-PR_wt_ (PDB ID: 4HLA) and GRL142- PR_wt_ (PDB ID: 5TYS) complexes had been determined previously^8,14^. The crystal structures of these four complexes were analyzed and compared with the polar and non-polar interactions. The polar interactions with PR_p51_ and PR_wt_ are shown in Fig. 1 and supplementary Fig. 1, respectively. The crystal structure of DRV- PR_p51_ complex revealed that the *bis*-THF moiety had polar interactions with Asp29 and Asp30 in the S2 site of protease (Fig. 1a). The hydroxyl group had polar interactions with the catalytic aspartates Asp25 and Asp25′, the -NH group had a hydrogen bond interaction with Gly27, and the aminobenzene moiety of DRV had a hydrogen bond interaction with Asp30′ present in the S2′ site. The carbonyl and sulfonyl moieties of DRV had polar interactions with the Ile50 and Ile50′ residues present in the protease flap through the tetracoordinated structural water molecule. GRL142 differed from DRV in having a crown-THF as the P2-moiety, a difluoro benzyl as the P1-moiety, and a cyclopropyl-amino-benzothiazole (Cp-Abt) as its P2′ moiety. In the GRL142-PR_p51_ complex, the crown-THF had polar interactions with Asp29 and Asp30, and the Cp-Abt moiety had polar interactions with Asp30′ (Fig. 1b). The structural moieties that are common between DRV and GRL142 also had polar interactions with the same protease residues of PR_p51_.

**Fig. 2:**
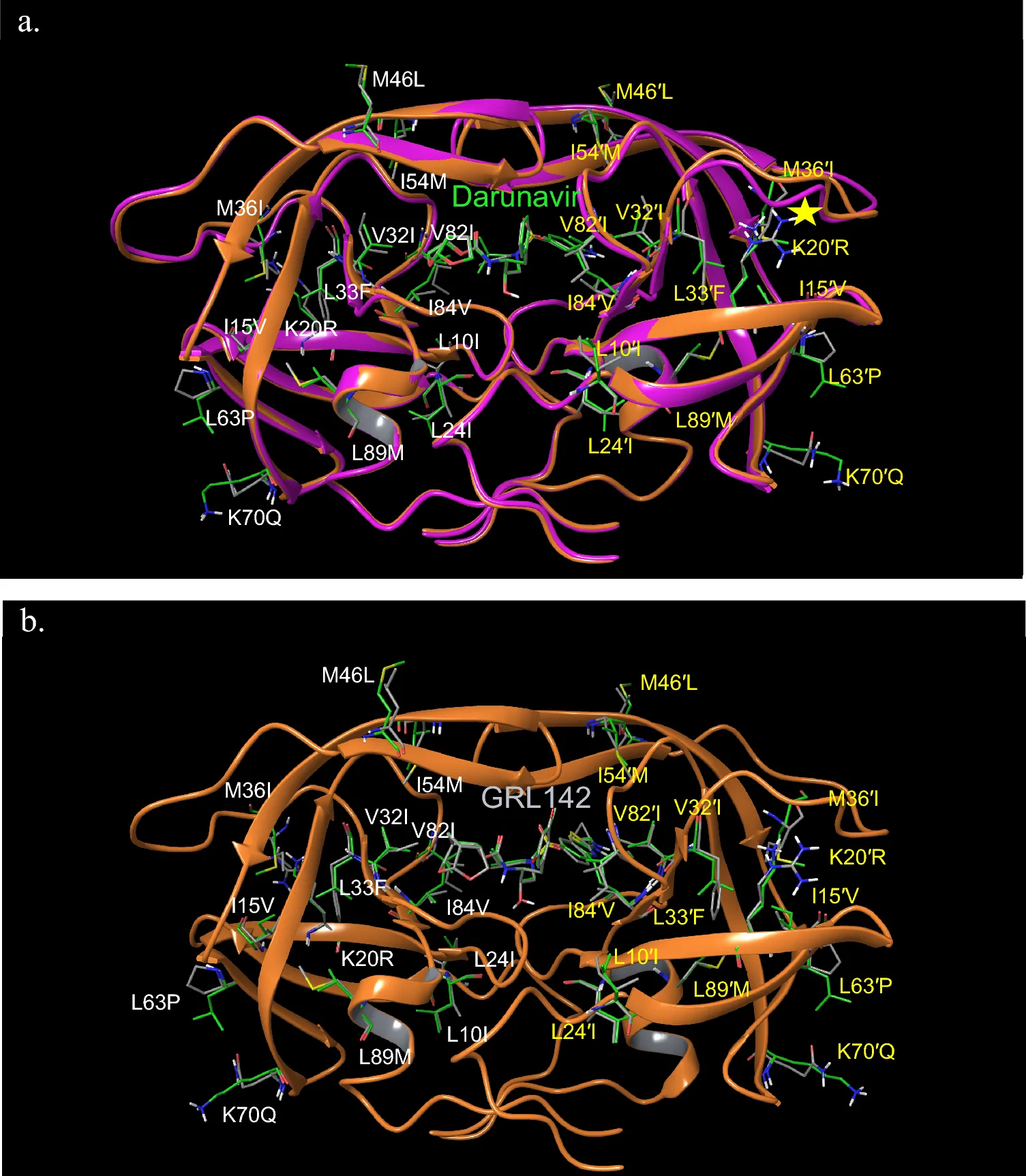
(**a**) Superimposition of the alpha carbons shows that the ribbon diagrams of DRV-PR_p51_ and DRV- PR_wt_ overlay well. DRV-PR_p51_ (gray carbons, purple ribbons) crystal structure is superimposed on the crystal structure of DRV-PR_wt_ (green carbons, orange ribbons). Overall, the ribbon diagrams of both structures superimpose very well. (**b.**) Superimposition of GRL142-PR_p51_ (gray carbons) and GRL142-PR_wt_ (green carbons) crystal structures show the differences in overlaps and conformations of the sidechains. While the backbone atoms overlap well, there are noticeable differences in the overlap of the sidechains of the substituted residues. The secondary structure of GRL142-PR_p51_ (orange ribbons) is shown.

Overall, the polar interactions of DRV and GRL142 with PR_p51_ were very similar to the polar interactions observed in their respective PR_wt_ complexes (supplementary Figs. 1a and b)^8,12^. The superposition of the alpha carbons of DRV-PR_p51_ complex on DRV-PR_wt_ is shown in Fig. 2a, and the secondary structures aligned well (Fig. 2a). However, there were differences in the conformations of the side-chains of the substituted residues (Fig. 2a). The same behavior was observed in the superposition of the PR_wt_ and PR_p51_ complexes of GRL142 (Fig. 2b). In summary, the overlays showed that the secondary structures had minimal differences, and not surprisingly most of the differences were in the conformation of the side-chains of the substituted residues (Fig. 2 a,b).

**Fig. 3:**
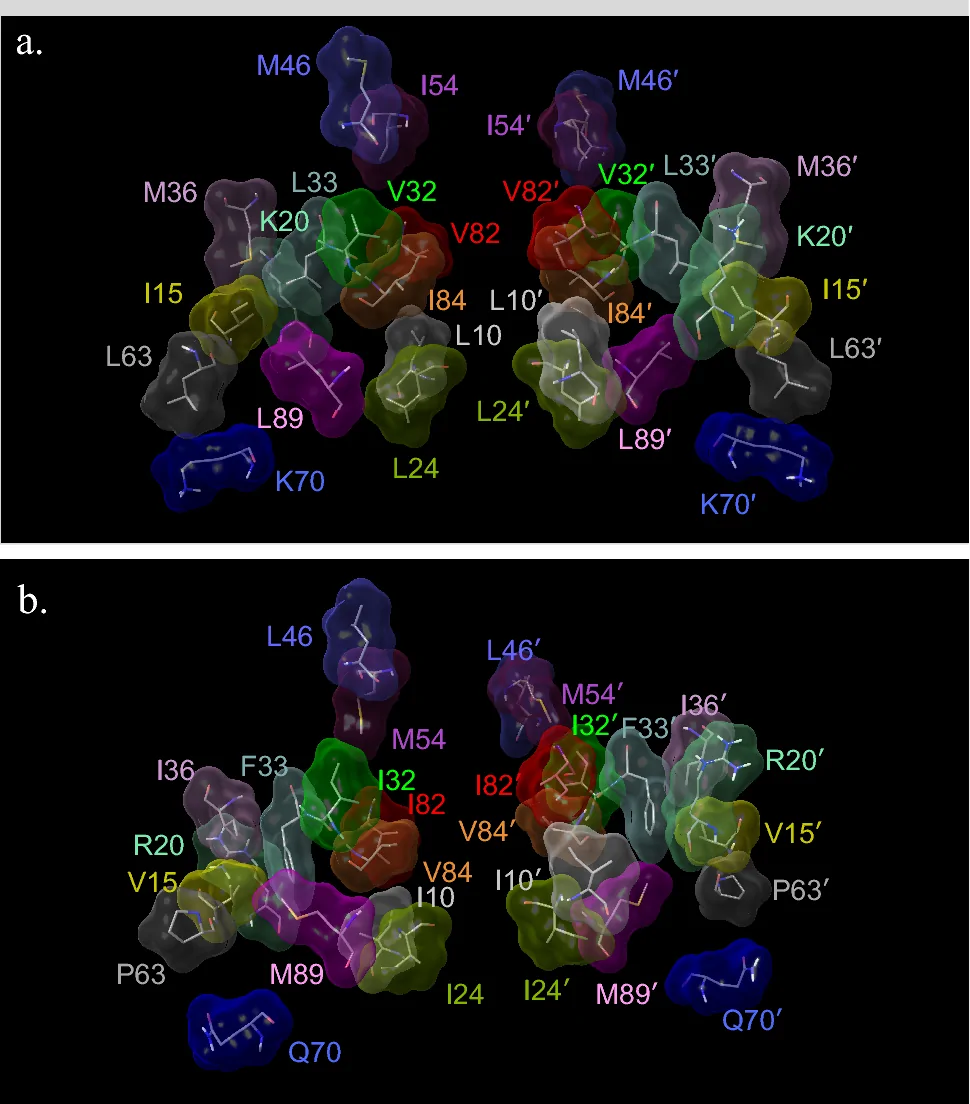
Molecular surfaces of residues undergoing substitution from PR_wt_ to PR_p51_ form clusters of interactions. The molecular surfaces of residues undergoing substitution for PR_wt_ (from DRV-PR_wt_ complex, panel a) and PR_p51_ (from DRV- PR_p51_ complex, panel b) are shown. The molecular surfaces of several residues have close interactions, and the nature of some of these interactions in PR_wt_ are different compared to those in PR_p51_. For example: Leu63/Lys70 of PR_wt_ (panel a) closely interact whereas Pro63/Gln70 of PR^p51^ (panel b) do not; The residue substitutions and surface colors are as follows: Leu10Ile (gray), Ile15Val (yellow), Lys20Arg (aquamarine), Leu24Ile (yellowish green), Val32Ile (green), Leu33Phe (turquoise), Met36Ile (plum), Met46Leu (light blue), Ile54Met (maroon), Leu63Pro (gray), Lys70Gln (blue), Val82Ile (red), Ile84Val (orange), Leu89Met (magenta).

The residues in the active site of DRV-PR_wt_ complex are shown in supplementary Fig. 2-a. We analyzed the van der Waals (vdW) surface interactions of the inhibitors with PR_wt_ and PR_p51_. The interactions of DRV with selected PR_wt_ residues are shown in supplementary Fig. 2-b. The nature of vdW contacts of DRV with PR_wt_ and PR_p51_ was mostly similar except for the following minor differences: the vdW surfaces of Ile32, Ile32′, and Ile47′ in DRV- PR_p51_ were farther away from DRV than were the vdW surfaces of Val32, Val32′, and Ile47′ in DRV- PR_wt_ (Fig. 3, supplementary Fig. 2-c and -d). Besides the residues that formed the active site of DRV- PR_wt_, two additional residues Arg8 and Lys45′ also formed a part of the active site in GRL142- PR_wt_ complex because of the interactions of these residues with the P2′ cyclopropyl group of GRL142 (PDB ID: 5TYS)^8^. Considering that the sidechain of Lys45’ has a higher B-factor, we also analyzed the trajectories from the MD simulation. The analyses demonstrated that the P2’ cyclopropyl group of GRL142 fluctuates between Arg8 and Lys45’- as would be expected for these interacting moieties (supplementary Fig. 3 a-f). There were minor differences in the vdW contacts of GRL142 with residues 29′, 32, 32′, 50, 50′,84, and 84′ in GRL142-PR_p51_ over GRL142-PR_wt_. In summary, in their respective crystal structures, there were no noteworthy differences in polar and van der Waals interactions of DRV-PR_wt_ over DRV-PR_p51_. The small differences did not explain the significant resistance of HIV_p51_ for DRV. The broad similarity of interactions also failed to satisfactorily explain the different resistance profiles of DRV and GRL142 against PR_p51_ and HIV_p51_ (Table 1).

**Changes in selected residue distances in DRV-PR**_**p51**_** from DRV-PR**_**wt**_** and in GRL142-PR**_**p51**_** from GRL142-PR**_**wt**_** highlight structural changes.** In the four crystal structures (DRV-PR_wt_, DRV-PR_p51_, GRL142-PR_wt_, and GRL142-PR_p51_), we measured distances between selected residues to determine if certain residues were closer or farther away from each other in the PR_p51_ complex compared to their distance separation in the corresponding PR_wt_ complex (supplementary Fig. 4). For example, the distance between the alpha carbons of Val15 and Phe33 of DRV-PR_p51_ is 9.62 Å compared to the distance of 10.26 Å between the alpha carbons of Ile15 and Leu33 of DRV-PR_wt_ indicating that these two residues are closer to each other in the DRV-PR_p51_ complex (supplementary Fig. 4, Left Column, 1^st^ Line). The distances between the alpha carbons of selected residues in DRV-PR_p51_ and GRL142-PR_p51_ were subtracted from the corresponding distances in their respective PR_wt_ complexes. The result is presented in Table 2. We found that these distances captured the concerted changes in the secondary structure of the PR_p51_ complexes over the PR_wt_ complexes. In fact, we observed changes and trends in inter-residue distances as follows: (i). For the protease monomer, the distal residues within the monomer came closer to each other in DRV-PR_p51_ over DRV- PR_wt_ (Table 2, Fig. 2a, supplementary Fig. 4). For example, in DRV-PR_p51_, Val15′ and Phe33′ were closer to each other by 0.82 Å compared to the distance between Ile15′ and Leu33′ in DRV- PR_wt_ (please see footnote in Table 2 to see the method). (ii). For the protease dimer, there was an increase in the distance between active site residues for DRV-PR_p51_ over DRV- PR_wt_. For example, the distance between Ile32 to Ile32′ in DRV-PR_p51_ is longer by 0.84 Å than the distance between Val32 to Val32′ in DRV- PR_wt_.  (iii). Within each monomer, the distance between an active site and a flap residue increased for DRV-PR_p51_ over DRV- PR_wt_. In DRV-PR_p51_, Ile32 and Leu46 were farther by 1.01 Å than the distance between Val32 and Met46 in DRV- PR_wt_. Overall, compared to DRV complexes, there was less structural perturbation between the structures of GRL142-PR_p51_ and GRL142- PR_wt_.

**Table 2:** Molecular dynamics simulations show the concerted changes in selected residue distances in the crystal structures of DRV-PR_p51_ over DRV-PR_wt_ and of GRL142-PR _p51_ over GRL142-PR_wt_. The distal residues come closer in PR _p51_ complexes compared to their distances in PR_wt_ complexes; the distance between two active site residues, and the distance between an active site residue and a flap residue increases from PR_wt_ complexes to PR_p51_ complexes.

Residue numbers	DRV-PR_wt_ to DRV-PR_p51_	GRL142-PR_wt_ to GRL142-PR _p51_	Description
15–33	0.64	0.59	These inter-residue distance differences measured changes distal from the active site within a monomer for the PR_wt_ and PR _p51_ complexes. A positive number indicated that these distal residues were closer to each other in the PR_p51_ complexes compared to their distances in the PR_wt_ complex
15’-33′	0.82	0.58	
15–36	1.65	1.83	
15’-36′	1.76	1.81	
20–33	0.63	0.38	
20′-33′	0.73	0.32	
20–36	1.52	1.55	
20′-36′	1.51	1.60	
32–32′	− 0.84	− 0.34	These residues are in the active site and measured inter-residue distances in the dimer. The negative numbers indicated that PR_p51_ complexes had longer distances between these active site residues over PR_wt_ complexes suggesting binding cavity expansion. GRL142-PR_wt_ to GRL142-PR_p51_ had a smaller expansion (smaller negative numbers) in binding cavity compared to DRV-PR_wt_ to DRV-PR_p51_
32′-33	− 0.92	− 0.53	
32′-82	− 0.65	− 0.16	
33–33′	− 0.87	− 0.71	
32–46	− 1.01	− 0.76	These measured distance changes between an active site and a flap residue within the same monomer. The negative numbers indicated that the PR_p51_ complexes had longer distances between these residues over PR_wt_ complexes suggesting cavity expansion
32′-46′	− 0.92	− 0.73	
32–54	− 0.63	− 0.47	
32′-54′	− 0.87	− 0.59	
33′-54′	− 0.83	− 0.57	
46–82	− 0.73	− 0.71	
46′-82′	− 0.67	− 0.71	

The distance between the alpha carbons of residues (column 1) in the PR_p51_ crystal structure of DRV or GRL142 is subtracted from the corresponding distance in the PR_wt_ crystal structure. A positive distance difference number (columns 2 and 3 for DRV and GRL142, respectively) indicated that the residues were closer to each other in the PR_p51_ complex than they were in the PR_wt_ complex; a negative distance difference number indicated that the residues were farther apart from each other in PR_p51_ over the PR_wt_ complex.

**The molecular surfaces of residues undergoing substitution from DRV-PR**_**wt**_** to DRV-PR**_p51_ **form clusters of interactions.** Both in in vitro and in clinical settings, DRV is known to have a high genetic barrier against HIV-1’s development of resistance^9,12,15^. In an attempt to generate HIV-1’s resistance against DRV, we had carried out resistance selection experiments using a mixture of 8 clinical HIV-1 variants that already had 9 to 14 PI-resistance-associated amino acid substitutions in protease^12^. Viral passage of this mixture of HIV-1 variants over 51 passages (one passage is seven to ten days) produced new variants of HIV-1 with significant resistance against DRV. HIV_p51_ had fourteen amino acid substitutions in the protease region (PR_p51_)^12^. Out of these fourteen amino acids, only two residues Val82 and Ile84 had tight van der Waals (vdW) surface interactions, and Val32 had moderate vdW interactions with DRV as observed in the crystal structure of the DRV-PR_wt_ complex^14,16^. The other eleven residues that underwent substitutions did not have a direct binding interaction with DRV. We generated the molecular surfaces of all fourteen residues that underwent substitution. It was intriguing that several of these residues spanning different structural domains of the protease dimer had close molecular surface interactions with one another (Fig. 3). For example, residues Ile15, Lys20, Leu33 and Met36 in DRV-PR_wt_ formed a cluster of vdW interactions (Fig. 3a, supplementary Fig. 4). Though Val15, Arg20, Phe33 and Ile36 in DRV-PR_p51_ also formed a cluster of residue surface interactions (Fig. 3b), there were differences in the surface contact areas. While Leu63 and Lys70 of DRV-PR_wt_ had close surface interactions, the surfaces of substituted Pro63 and Gln70 in DRV-PR_p51_ did not interact (Fig. 3a and b). There was a noticeable difference in the interactions between Val82 and Leu10 of DRV-PR_wt_ (which did not interact), compared to the much closer proximity of Ile82 to Ile10 in the DRV-PR_p51_ (Fig. 3a and b, supplementary Fig. 4). One could infer that structural rearrangements in the crystal structure of DRV-PR_p51_ made residues Ile10 and Ile82 move closer. Other noteworthy changes in residue surface interactions between DRV-PR_wt_ and DRV-PR_p51_ were observed for the V32I-L33F-V82I-I84V, I15V-L63P-K70Q, and V32I-I54M substitutions. (supplementary Fig. 4). In summary, the residues undergoing substitution not only formed several clusters of surface interactions, but also the nature of these interactions differed between DRV-PR_wt_ and DRV-PR_p51_. Mostly hydrophobic residues were involved in the substitution, with isoleucines being present eight times either as a wild-type or as a mutant residue. Eleven substitutions have either an Ile or Val present, and no residue other than Ile, Leu or Val is present in six substitutions (L10I, I15V, L24I, V32I, V82I, I84V). Foulkes-Murzycki et al*.* and Ishima et al*.* also observed the predominant involvement of hydrophobic residues in drug-resistant HIV protease^17,18^. The core hydrophobic residues mutate to other hydrophobic residues. The hydrophobic contacts are important for protease structure and function and are called collectively “hydrophobic sliding”. Most of the significant hydrogen bond interactions are maintained within the core. There are many isoleucines in the hydrophobic core, and the methyl groups of Ile have more possible conformations. Many of the distal residues undergoing substitution are hydrophobic in nature. They identified nineteen residues, many of them Leu and Ile that form the hydrophobic core of HIV protease^17,18^. Mutation of these residues changes the vdW interactions with negligible energetic penalties.

## Molecular dynamics (MD) simulation gives insight into the structural fluctuations

- Analysis of inter-residue distance changes from MD shows the extent of structural changes

We carried out MD simulations of DRV-PR_wt_, DRV-PR_p51_, GRL142-PR_wt_, and GRL142- PR_p51_ complexes for 1500 nano seconds (ns). In the simulation, the distances between residues fluctuated over time. At intervals of 0.5 ns, we calculated the relative distance changes between the alpha carbons of selected residues with respect to the corresponding distances observed in the crystal structures of the respective DRV-PR_wt_ or GRL142- PR_wt_ complexes (Table 3, supplementary Fig. 5). Column numbers 1 and 2 list the PR_wt_ and PR_p51_ residues, respectively, whose inter-residue distances were measured. For example, row # 1 measured relative distances between Ile15 and Leu33 for PR_wt_ and between Val15 and Phe33 for PR_p51_ complexes. Column # 3 and beyond list the relative closest and farthest distances between these residues, as determined from MD, compared to the respective distances measured in the wild-type crystal structure complexes. We illustrate the calculation of relative closest and farthest distances taking the example of the separation between Val32 and Val82′ for DRV-PR_wt_ and between Ile32 and Ile82′ for DRV-PR_p51_ (Table 3, row #9). The distance between Val32 and Val82′ in the DRV-PR_wt_ crystal structure was 18.38 Å (supplementary Fig. 5a). The closest and farthest distances between Val32 and Val82′ from the MD simulation were 16.33 Å and 20.51 Å, respectively (Sup. Figure 5a). The difference of these distances from the crystal structure distance of 18.38 Å resulted in relative closest and farthest distances of -2.05 Å and 2.13 Å, respectively, for DRV-PR_wt_ (Table 3, row #9, column #3 and #4). From the MD simulation of DRV-PR_p51_, the closest and farthest distances between Ile32 and Ile82′ were 16.79 Å and 21.97 Å respectively (supplementary Fig. 5b), resulting in relative closest and farthest distances of -1.59 Å and 3.59 Å, respectively (Table 3, row #9, column #5 and #6).

**Table 3:** Farthest and closest relative distances (Å) between selected residues from molecular dynamics simulation quantify inter-residue fluctuations.

Column #	1	2	3	4	5	6	7	8	9	10
Row #	Residues (PR^wt^)	Residues (PR^p51^)	Closest Distance (Å) (DRV-PR^wt^)	Farthest distance (Å) (DRV-PR^wt^)	Closest Distance (Å) (DRV-PR^p51^)	Farthest Distance (Å) (DRV-PR^p51^)	Closest Distance (Å) (142-PR^wt^)	Farthest distance (Å) (142-PR^wt^)	Closest Distance (Å) (142-PR^p51^)	Farthest Distance (Å) (142-PR^p51^)
1	Ile15-Leu33	Val15-Phe33	− 1.45	2.04	− 2.54	2.61	− 1.48	2.67	− 1.49	2.85
2	Ile15′-Leu33′	Val15′-Phe33′	− 2.54	1.68	− 2.22	1.80	− 1.37	2.25	− 3.23	2.01
3	Asp25-Asp25′	Asp25-Asp25′	− 0.93	0.53	− 1.02	0.59	− 1.24	0.72	− 1.41	0.21
4	Asp29-Ile47	Asp29-Ile47	− 0.92	4.27	− 0.73	3.29	− 0.90	2.80	− 0.15	3.17
5	Asp29′-Ile47′	Asp29′-Ile47′	− 1.41	3.30	− 0.89	3.72	− 1.20	3.38	− 0.56	3.01
6	Val32-Val32′	Ile32-Ile32′	− 2.29	1.31	− 1.87	1.39	− 2.20	0.95	− 2.74	0.72
7	Val32-Ile54	Ile32-Met54	− 1.36	2.27	− 0.85	2.69	− 1.12	2.70	− 0.69	2.47
8	Val32′-Ile54′	Ile32′-Met54′	− 1.54	2.45	− 1.17	2.58	− 1.10	2.54	− 1.07	1.96
9	Val32-Val82′	Ile32-Ile82′	− 2.05	2.13	− 1.59	3.59	− 1.68	2.70	− 2.25	3.40
10	Val32-Ile84′	Ile32-Val84′	− 2.10	1.22	− 1.84	1.23	− 1.76	1.46	− 2.39	0.72
11	Val32′-Ile84	Ile32′-Val84	− 2.03	1.37	− 1.83	0.97	− 2.21	1.14	− 1.94	1.35
12	Ile50-Ile50′	Ile50-Ile50′	− 0.92	2.26	− 0.86	2.94	− 0.94	1.90	− 1.01	0.78
13	Ile50-Thr80	Ile50-Thr80	− 1.56	3.05	− 1.88	4.69	− 1.36	2.27	− 1.78	3.03
14	Ile50′-Thr80′	Ile50′-Thr80′	− 1.77	3.19	− 1.38	3.44	− 1.50	2.08	− 1.34	2.77
15	Leu63-Lys70	Pro63-Gln70	− 1.98	1.35	− 1.90	1.49	− 2.19	1.30	− 1.76	1.44
16	Leu63′-Lys70′	Pro63′-Gln70′	− 1.70	1.26	− 1.54	1.53	− 1.18	1.24	− 1.61	1.42
17	Leu63-Leu89	Pro63-Met89	− 1.34	3.55	− 1.20	1.55	− 1.49	2.90	− 1.41	1.22
18	Leu63′-Leu89′	Pro63′-Met89′	− 1.51	2.82	− 1.24	1.53	− 1.84	1.95	− 1.58	1.32
19	Val82-Val82′	Ile82-Ile82′	− 2.19	2.75	− 1.26	4.10	− 2.30	2.40	− 1.06	4.11
20	Ile84-Ile84′	Val84-Val84′	− 1.84	1.33	− 1.69	1.05	− 1.67	1.47	− 1.62	1.11

Column 1 (PR^wt^) and column 2 (PR_DRV_^R^_p51_, abbreviated as PR^p51^) list the residues for which distances are measured. Closest and farthest relative distances between the alpha carbons of residues from MD is calculated relative to the corresponding distance observed in the crystal structure of the respective DRV-PR^wt^ or 142-PR^wt^ complex. For example, the distance between Val32 and Val82′ in the DRV-PR^wt^ crystal structure is 18.38 Å; closest and farthest distances between these residues from MD are 16.33 Å and 20.51 Å respectively (Sup. Figure 1a). The relative closest and farthest distances between these residues in DRV-PR^wt^ (row # 9, columns 3 and 4) is -2.05 Å (16.33 Å -18.38 Å) and 2.13 Å (20.51 Å -18.38 Å), respectively. In the MD simulation of DRV-PR_DRV_^R^_p51_, the closest and farthest distances between I32 and I82′ are 16.79 Å and 21.97 Å respectively (Sup. Figure 1b), resulting in relative closest and farthest distances (row # 9, columns 5 and 6) of -1.59 Å (16.79 Å-18.38 Å) and 3.59 Å (21.97 Å-18.38 Å), respectively. A negative relative distance indicates that the residues are closer compared to the distance in the wild-type crystal structure and a positive relative distance indicates that they are farther apart.

In the MD simulation of DRV-PR_p51_ complex, the closest and farthest distances between the alpha carbons of Ile50 and Thr80 were 9.50 Å and 16.07 Å, respectively (supplementary Fig. 5c). Compared to the distance observed between these residues in the DRV-PR_wt_ crystal complex (11.38 Å), the relative closest and farthest distances between Ile50 and Thr80 from the MD simulation of DRV-PR_p51_ complex were -1.88 Å and 4.69 Å, respectively (Table 3, row #13, columns #5 and #6). Following the same protocol (Sup. Figure 5d), we calculated the relative closest and farthest distances between Ile50 and Thr80 for GRL142-PR_p51_ (Table 3, row #13, columns #9 and #10), showing -1.78 and 3.03, respectively.

For DRV-PR_wt_ complex, the flap residues Ile50 and Ile50′ were closest by -0.92 Å and farthest by 2.26 Å (Table 3, row #12, columns #3 and #4). For GRL142- PR_wt_ complex, the flaps were closest by a similar distance (-0.94 Å) and farthest apart by 1.90 Å (Table 3, row #12, columns #7 and #8). Comparing the farthest Ile50-Ile50′ relative distance separations observed in MD simulations, the flaps of GRL142- PR_wt_ complex remained in a slightly more closed conformation than the flaps of DRV-PR_wt_ complex. Ile50 and Ile50′ were farthest by 2.94 Å for DRV-PR_p51_ compared to only 0.78 Å for GRL142-PR_p51_ (Table 3, row #12, columns #6 and #10), suggesting that the flaps of DRV-PR_p51_ had larger separation while GRL142-PR_p51_ remained in a closed flap conformation throughout the duration (1500 ns) of the simulation. When Ile50 and Ile50′ were farthest apart (by 2.94 Å) in DRV- PR_p51_, DRV had a decrease of vdW interactions with Ile32, Ile32′, Gly48, Gly49, Pro81′, Ile82, and Val84.

The distance between Ile50 and Thr80 (and between Ile50′ and Thr80′) is a measure of the dynamical changes in HIV protease, and its influence on binding interactions with inhibitors. Thr80 and Thr80′ are part of the so-called 80s loop and nearby residues Pro81′, Val82, Val82′, Ile84, and Ile84′ had direct vdW interactions with DRV and GRL142. The farthest relative distance between Ile50-Thr80 was 4.69 Å for DRV-PR_p51_ compared to 3.05 Å for DRV-PR_wt_, indicating that there was a larger separation (of 1.64 Å) for DRV-PR_p51_ (Table 3, row # 13, column # 4 and 6). In contrast, the farthest relative distance between Ile50-Thr80 for GRL142-PR_wt_ was 2.27 Å, and it increased to 3.03 Å for GRL142-PR_p51_ (Table 3, row #13, columns #8 and #10). This was a smaller perturbation (by 0.76 Å) compared to that observed for DRV-PR_p51_. The larger structural perturbations had a direct influence on binding interactions. For example, Val82 and Ile84 had tight vdW interactions with DRV in the DRV-PR_wt_ complex; however, when Ile50 and Thr80 were farthest apart in DRV-PR_p51_, the isopropyl group of DRV lost vdW interactions with Ile82 and Val84. Another example is when Ile82 and Ile82′ were farthest apart in DRV-PR_p51_ (relative distance separation of 4.10 Å), DRV had reduced van der Waals contact not only with Ile82, but also with Arg8′, Ile47, Ile47′, Gly48, Gly48′, Gly49, Gly49′, Ile50′, and Pro81′.

Not every inter-residue distance of PR_wt_ underwent significant changes in PR_p51_ in the MD simulation. The relative closest distances between Asp25 and Asp25′ for DRV-PR_wt_ and DRV-PR_p51_ were similar, and so were the relative farthest distances (Table 3, row # 3, column #s 3–6). This was not surprising considering the structural region that the catalytic aspartates occupy.

The closest and farthest distances need to be analyzed along with the population distribution of the relative distances. A higher population of longer distances between residues important for inhibitor binding is likely to have a larger effect on reducing the binding affinity. We analyzed the relative population at different distance intervals from the mean distance observed in the wild-type crystal structures below.

Comparison of columns 4 and 8 indicated the maximum structural perturbations associated with DRV-PR_wt_ over GRL142-PR_wt_. The farthest distances between Ile50-Ile50′, Ile50-Thr80, Ile50′-Thr80′, Asp29-Ile47, Val32-Val32′ and Val32-Val82′ were longer (between 0.36 Å to 1.47 Å) for DRV-PR_wt_ complex compared to GRL142-PR_wt_ complex. This result suggested that the binding cavity for GRL142-PR_wt_ complex was more compact compared to the binding cavity for DRV-PR_wt_ complex and provided another explanation for the higher binding affinity of GRL142 for PR_wt_ (Table 1).

**Fig. 4:**
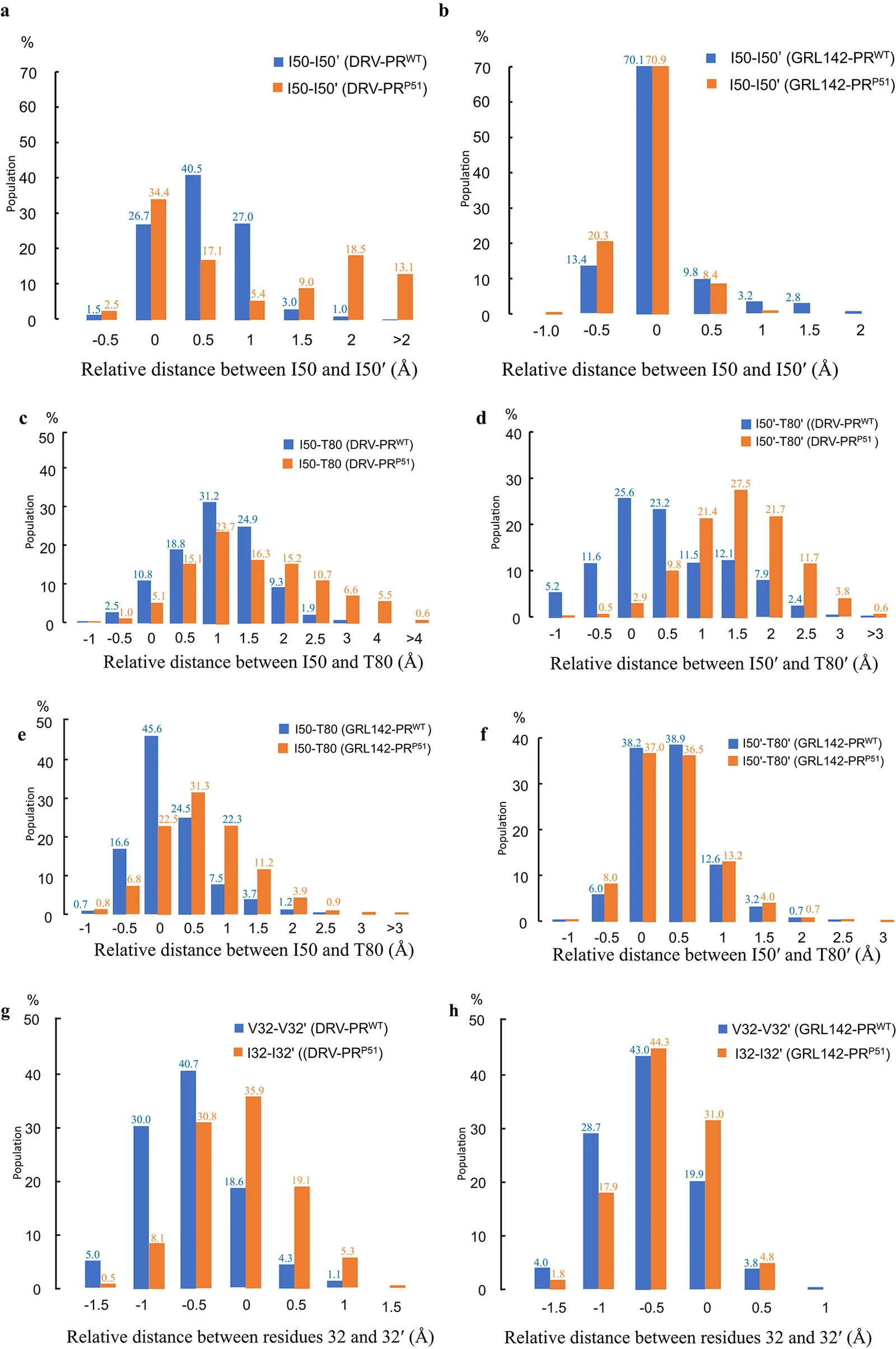
Molecular dynamics (MD) shows that DRV-PR_51_ has a larger population of structures farther than the equilibrium distance observed in the wild-type crystal structure. Panel a: In the MD simulations of DRV-PR_wt_ and DRV-PR_p51_, about 4% and 41% of the respective populations had Ile50-Ile50’ distance longer than 1 Å compared to the distance observed in the DRV-PR_wt_ crystal structure. Panel b: For GRL142-PR_wt_ and GRL-PR_p51_, about 3% and 1% of the respective populations had Ile50-Ile50’ distance longer than 1 Å compared to the distance observed in the GRL-PR_wt_ crystal structure. Panel c: For DRV-PR_wt_ and DRV-PR_p51_, 2% and 23% of the respective populations had Ile50 to Thr80 distance longer than 2 Å compared to the distance observed in the DRV-PR_wt_ crystal structure. Panel d: For DRV-PR_wt_ and DRV-PR_p51_, 3% and 16% of the respective populations had Ile50′ to Thr80′ distance longer than 2 Å compared to the distance observed in the DRV-PR_wt_ crystal structure. Panel e: For GRL142-PR_wt_ and GRL-PR_p51_, about 1% and 5% of the respective populations had Ile50′ to Thr80′ distance longer than 2 Å compared to the distance observed in the GRL142-PR_wt_ crystal structure. Panel f: There is very little difference in population distributions for GRL142-PR_wt_ and GRL-PR_p51_ for the Ile50′ to Thr80′ distances. There is no number label for population that is less than 0.5%.

The farthest distance between certain residues increased substantially for DRV-PR_p51_ over DRV-PR_wt_. The relative farthest distance between Val32-Val82′ for DRV-PR_wt_ was 2.13 Å, and the distance between these residues increased by 1.46 Å to 3.59 Å for DRV-PR_p51_ (Table 3, row 9, columns #4, #6). The farthest distance between residues 82 and 82′ increased from 2.75 Å for DRV-PR_wt_ to 4.10 Å for DRV- PR_p51_ (Table 3, row 19, columns #4, #6), and suggested that the binding cavity increased for DRV- PR_p51_.

b. Analysis of population distribution at relative distance intervals
i. Changes in flap separation:

HIV-1 protease has two molecular “flaps” which move a distance of up to ~ 7 Å when the enzyme becomes associated with a substrate^19^. For acquiring PI resistance, the virus uses a strategy to alter the conformation and movement of the two flaps, causing them to shift between open, semi-open, and closed conformations. Assuming that the distance between Ile50 and Ile50’ is a measure of the separation between the protease flaps, we first compared the changes in relative distance between Ile50 and Ile50’ (Fig. 1-a and -b). The protease flaps are in a closed conformation in the crystal structures of DRV-PR_wt_ and GRL142-PR_wt_ complexes (Fig. 1-a and -b). From the MD simulations, we compared the changes in population with relative distances between Ile50 and Ile50’. Figure 4a shows that a significantly higher population (~ 36% more) of structures had Ile50-Ile50’ relative distance longer than 1 Å for DRV-PR_p51_ compared to DRV-PR_wt_, with nearly 13% more structures with Ile50-Ile50’ relative distances longer than 2 Å for DRV-PR_p51_. In contrast, the population distributions at different distance separations between Ile50-Ile50’ were very similar for GRL142-PR_wt_ and GRL142-PR_p51_ complexes (Fig. 4b). In summary, in the MD simulation, the flaps in GRL142-PR_p51_ stayed in conformations similar to the flaps of GRL142-PR_wt_, whereas the flaps of DRV-PR_p51_ had a higher population of structures with wider separation between Ile50 and Ile50’ compared to the DRV-PR_wt_ complex. These data strongly suggest that DRV-PR_p51_ had more semi-open conformations compared to the wild-type complex and that the active-site cavity in PR_p51_ is expanded than that in PR_wt_, thereby resulting in the acquisition of viral resistance to DRV.

ii. Population analysis of Ile50-Thr80 distance:

We also analyzed the population distribution at different relative distance intervals between Ile50 and Thr80 since the distance between these two residues affects the conformation around the active site and the protease flaps (Fig. 4 c-f). The Ile50 to Thr80 distance was longer than the equilibrium crystal structure distance by more than 1.5 Å in only 12% of the structures for DRV-PR_wt_ compared to 39% of the population for DRV-PR_p51_. Only 1% and 5% of structures had Ile50 to Thr80 distance longer than 1.5 Å for GRL142-PR_wt_ and GRL142-PR_p51_, respectively. Thus, not only was there a higher population of structures with longer Ile50-to-Thr80 distances for DRV-PR_wt_ over GRL142-PR_wt_, but there was also a significantly higher population with longer Ile50-to-Thr80 distances for DRV-PR_p51_ compared to GRL142-PR_p51_. Similarly, 27% more structures had longer (> 1.5 Å) Ile50′-to-Thr80′ distance for DRV-PR_p51_ over DRV-PR_wt_, while there were no significant differences in the population of Ile50′-to-Thr80′ relative distances between GRL142-PR_wt_ and GRL142-PR_p51_.

These differences in population between DRV-PR_wt_ and GRL142-PR_wt_ should at least in part explain the higher potency of GRL142 over DRV against HIV_wt_. Also, the significantly higher population of longer Ile50-to-Thr80 and Ile50′-to-Thr80′ distances for DRV-PR_p51_ likely impacts the conformation of 80 s loop and binding interactions of DRV.

Moreover, we analyzed the changes in population distribution at different relative distance intervals for other important inter-residue distances. The changes in population distribution with relative distance separations between Val32 and Val32′ (PR_wt_ complexes) and between Ile32 and Ile32′ (PR_p51_ complexes) are shown in (Fig. 4 g-h). Nearly 95% of the population had V32 to Val32′ relative distance no longer than the equilibrium crystal structure distance for DRV-PR_wt_, whereas only 75% of the population had Ile32 to Ile32′ distance no longer than the equilibrium crystal structure distance for DRV-PR_p51_. In comparison, nearly 95% of the population had V32 to Val32′ (GRL142-PR_wt_) and Ile to Ile32′ (GRL142-PR_p51_) relative distances that were not longer than the equilibrium crystal structure distance of GRL142-PR_wt_. While there was not a large distance separation between residues 32 and 32′ in the MD of the four complexes, only DRV-PR_p51_ generated a population of structures (~ 5%) that had relative distances greater than 0.5 Å; the other structures generated population distributions of less than 0.5% in that distance range.

c. Active site volumes.

**Fig. 5:**
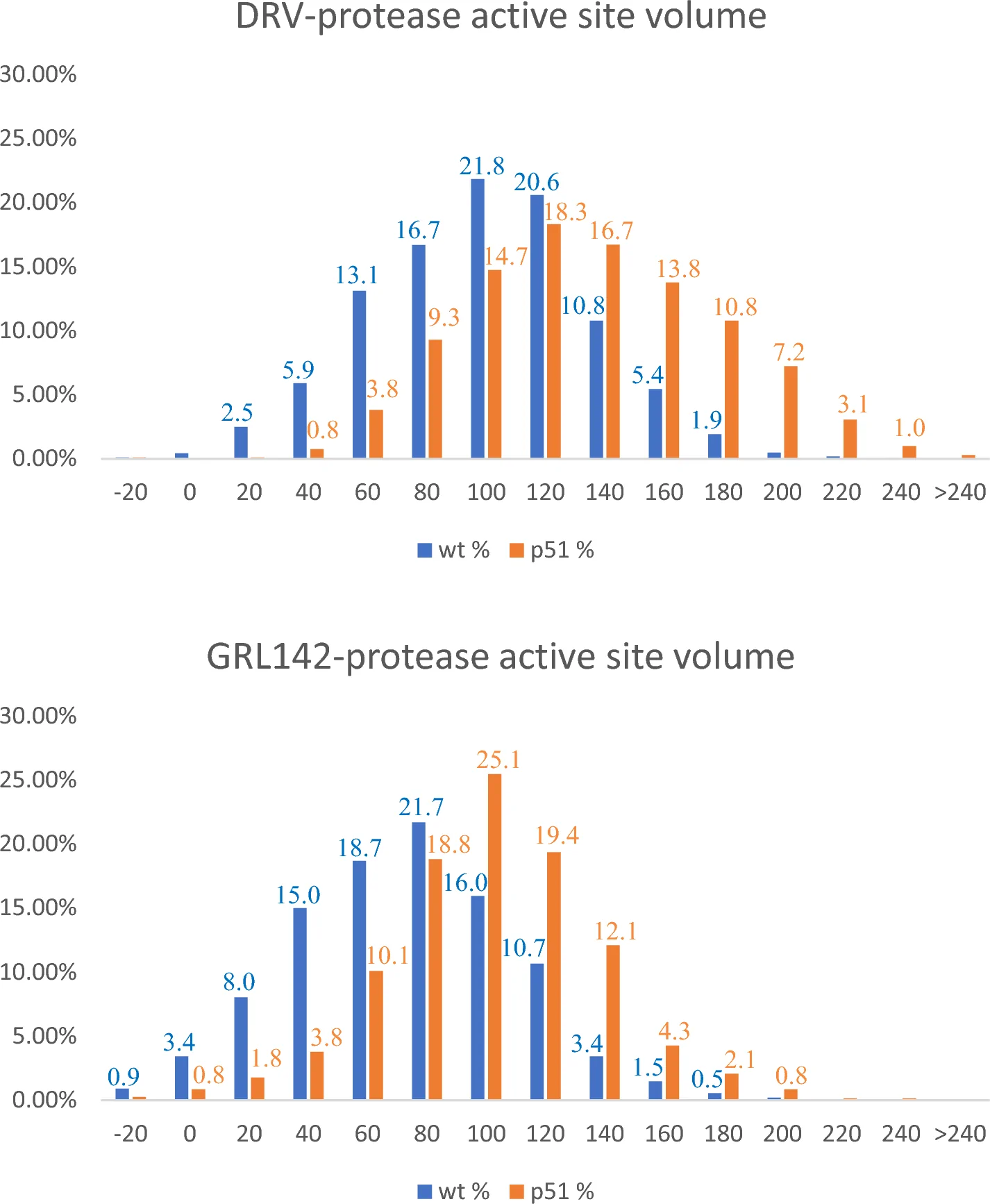
Calculation of active site volumes from MD simulation trajectories shows that the active site of DRV-PR_p51_ undergoes a larger expansion compared to GRL142-PR_p51_. Panels a-b: 61% of the DRV-PR_wt_ and 84% of GRL142-PR_wt_ population has active site volumes within 100 Å^3^ of the corresponding wild-type active site volumes computed from the crystal structures. In contrast, only 29% of the DRV-PR_p51_ population has an active site volume within 100 Å^3^ of the active site volume, whereas 61% of the GRL142-PR_p51_ population is within 100 Å^3^ of the crystal structure. The MD simulation analysis unequivocally demonstrates that the active site of DRV-PR_p51_ undergoes a larger expansion compared to the expansion of GRL142-PR_p51_ over GRL142-PR_wt_.

We computed the active site volumes of DRV and GRL142 with PR_wt_ or PR_p51_ with a python script from Schrödinger (https://learn.schrodinger.com/private/edu/release/2024-1/Documentation/html/utilities/program_utility_usage/trajectory_binding_site_volumes.html). The volume of the active site in DRV’s complex with PR_wt_ (PDB ID 4HLA) was 447.3 Å^3^. The active site volume for the DRV-PR_p51_ crystal structure was 484.0 Å^3^, an increase by 36.7 Å^3^(8.2%). The volume of the active site when GRL142 complexed with PR_wt_ was 496.7 Å^3^. This is larger than the active site volume of DRV- PR_wt_ complex but isn’t surprising considering that the active sites are dynamic and since GRL142 is a significantly larger molecule than DRV, and the protease active site undergoes an expansion to accommodate the larger ligand. The volume of the GRL142-PR_p51_ protease active site was 543.3 Å^3^, an increase by 46.6 Å^3^(9.4%). We then analyzed the active site volumes of these complexes by analyzing the MD simulation trajectories. In the MD simulation, 61% of the DRV-PR_wt_ and 84% of GRL142-PR_wt_ population has an active site volume within 100 Å^3^ of the corresponding wild-type active site volumes in the crystal structure. In contrast, only 29% of the DRV-PR_p51_ population has an active site volume within 100 Å^3^ of the DRV- PR_wt_ active site volume, whereas 61% of the GRL142-PR_p51_ population is within 100 Å^3^ of the volume in the crystal structure of GRL142-PR_wt_. These data strongly confirm that the MD simulation analysis unequivocally demonstrates that the active site of DRV-PR_p51_ undergoes a larger expansion compared to the expansion of GRL142-PR_p51_ over GRL142-PR_wt_. (Fig. 5).

## Discussion

Most PI-resistance-associated amino-acid substitutions deemed as major substitutions are proximal to the active site of the protease and cause changes in interactions with the PI. Examples of such major substitutions that are proximal include D30N for nelfinavir, V32I for atazanavir and lopinavir, V82A for atazanavir, lopinavir, and indinavir, I84V for many PIs including lopinavir, and tipranavir^10^. Though rare, a few distal substitutions such as L90M (for nelfinavir and saquinavir) are categorized as a major drug-resistant substitution^10^.

The role of distal mutations in HIV protease resistance is not clear but has been examined by using various experimental and computational techniques. Using a combination of enzymatic assays, crystallography, and MD simulations, Henes et al*.* studied the effect of distal mutations in HIV-1 protease in causing drug resistance^20^. They postulated that the effect of distal mutations somehow propagates throughout protease and impacts the active site.

Prediction of binding affinity of small molecules to their protein targets is an area of active research ^21–23^. HIV-1 protease flaps can stay in open, semi-open, curled or closed conformation. The conformation of the flaps must have a discernable effect on the geometry of the active site. For inhibitor-bound proteases including many drug-resistance-associated proteases, the flaps remain in a closed conformation. Moreover, the root mean square deviation of the overlays of the crystal structures of many drug-resistance-associated proteases are rather small. The change in interactions with amino-acid-substitutions is small in many instances – and doesn’t satisfactorily explain the observed changes in activity. Afterall, for a given protease-inhibitor combination, the change in geometry, volume and binding interactions with substitution(s) are significant contributors to the changes in binding affinity.

We studied the mechanism of resistance of HIV_DRV_^R^_p51_ (HIV_p51_) against DRV and GRL142. HIV_p51_ has three proximal and eleven distal substitutions in the protease (PR_p51_). X-ray crystal structures of DRV with PR_wt_ and PR_p51_ showed that the amino-acids undergoing substitution form clusters of vdW interactions and the surface contacts between residues are different between PR_wt_ and PR_p51_ complexes (Fig. 3). With MD simulations, we were able to demonstrate that the distance between residues in the protease dimer that defines the active site is longer than the equilibrium crystal structure distance in a larger population of structures. We showed that the so-called minor amino-acid substitutions, along with the major amino-acid substitutions increase the inter-residue distances defining the protease binding cavity and contribute to an increase in the active site volume. This must be contributing to the loss of binding affinity of DRV with PR_p51_ and the resistance of HIV_DRV_^R^_p51_ against DRV. Our MD simulation shows that the non-active site and active site substitutions work in concert to carry out significant structural changes in DRV-PR_p51_ over DRV-PR_wt_ complex. In comparison, the structural changes are less between GRL142-PR_wt_ and GRL142-PR_p51_ complexes.

Certain protease inhibitors such as DRV possess structural characteristics that make resistance relatively difficult to develop, and multiple protease mutations are generally required before clinically meaningful loss of susceptibility occurs. In the real-world setting, it is critical to delay the development of resistance-associated amino acid substitutions by reducing the generation of viral diversity itself, rather than simply suppressing viral replication. The insight gained from the analysis of our MD simulation can potentially be useful in deciding if switching to a DRV-containing therapy regimen is suitable for HIV-infected patients undergoing treatment with other clinically approved interventions. MD simulations of protease with drug-resistance-associated mutations can be carried out to determine if a particular set of mutations results in expansion of the active site and may cause resistance against DRV. In the present study, our MD simulations were able to differentiate the difference in resistance profile between DRV and GRL142. Thus, MD can potentially be used to predict the activity of newer inhibitors against drug resistant variants.

The distal substitutions are traditionally called minor substitutions and are deemed not to influence inhibitor binding. Our work demonstrates that the distal substitutions play an important part in the expansion of the active site, and presents a simple and intuitive explanation. The expansion of the active site resulted in loss of van der Waals interactions of DRV with active site residues. The results suggest that the minor substitutions have a more direct impact on the loss of binding of DRV and in causing HIV’s drug resistance against DRV. The study suggests reexamination of the structural and functional role of distal/minor substitutions seen in drug-resistance-associated HIV protease. It might be worthwhile exploring this in other antiviral drug targets of HIV, and in the resistance mechanism in other therapeutic areas.

## Materials and methods

**Cells, viruses, and antiviral agents.** MT-4 cells (RRID: CVCL_2632), authenticated using the STR profiling (ATCC, Manassas, VA), were grown in RPMI1640 culture medium supplemented with 10% fetal calf serum (Gemini Bio-Products) plus antibiotic–antimycotic (Anti-Anti; Gibco). HIV_DRV_^R^_p51_, a highly protease inhibitors (PIs)-/nucleoside reverse transcriptase inhibitors (NRTIs)-resistant laboratory generated HIV-1 variant, had previously been obtained in vitro^12^. GRL142 was designed and synthesized as previously described^24^. Darunavir (DRV) was purchased from Sigma-Aldrich.

**Drug susceptibility assay**. The susceptibilities of laboratory strains of a wild-type HIV-1_NL4-3_ (HIV_wt_), and a highly PIs/NRTIs-resistant HIV-1 variant (HIV_DRV_^R^_p51_) to DRV and GRL142 were determined as previously described^8,24^ with minor modifications. For p24 assay, MT-4 cells (5 × 10^5^/ml) were exposed to fifty 50% tissue culture infectious dose (TCID_50_) of each HIV-1 preparation in the absence or presence of various concentrations of each agent and were cultured at 37 °C. On day 7 of the culture, the supernatants were harvested and the amounts of p24 Gag protein were determined by using a fully automated chemiluminescent enzyme immunoassay system (Lumipulse G1200; Fujirebio). The drug concentrations that suppressed the production of p24 Gag protein by 50% (50% effective concentrations; EC_50_) were determined by comparing the average levels of p24 production in drug-free control cell cultures. All assays were performed in triplicate.

**Protein expression and purification.** PR_DRV_^R^_p51_ was purified as previously reported^8^. Briefly, Rosetta (DE3) pLysS strain (Novagen) was transformed with PR_DRV_^R^_p51_ expression vector^25^. The culture was grown overnight with Luria broth plus kanamycin and chloramphenicol (LB^Km+/Cp+^) at 37 ºC. The overnight culture was added to ZYM-10052 (1.0% N-Z amine, 0.5% yeast extract, 25 mM disodium hydrogen phosphate, 25 mM potassium dihydrogen phosphate, 50 mM ammonium chloride, 5 mM sodium sulfate, 1.0% glycerol, 0.05% glucose, 0.2% α-lactose, 2 mM magnesium sulphate) plus kanamycin and chloramphenicol (ZYM-10052^ km+/Cp+^). One litter of ZYM-10052^ km+/Cp+^ was used for PR_DRV_^R^_p51_. The ZYM-10052^ km+/Cp+^ culture was further continued at over 150 rpm at 37 ºC for 20 ~ 22 h^26^. Then the culture was spun down and pellets were resuspended in buffer A (20 mM Tris, 1 mM EDTA, and 1 mM DTT) and lysed with sonication. The cell lysates were centrifuged, and an inclusion body fraction was collected. The inclusion body fraction was washed five times with buffer A containing 2 M urea and then with buffer A without urea. The twice-washed pellet was solubilized and PR_DRV_^R^_p51_ was unfolded with 100 mM formic acid (pH 2.8)^27^. The unfolded PR_DRV_^R^_p51_ was purified using the fast protein liquid chromatography system (ÄKTA pure 25; GE Healthcare) and separated using the reverse phase chromatography column (RESOURS RPC 1 mL; GE Healthcare) using the gradient of buffer B (1.0% formic acid, 2.0% acetonitrile) and buffer C (1% formic acid, 70% acetonitrile). The collected fractions containing PR_DRV_^R^_p51_ was subjected to desalting (HiTrap Desalting; GE Healthcare) and the eluted solution was equilibrated using 100 mM formic acid and stored at − 80 °C until use.

**Enzyme inhibition assay.** The inhibitory activities of DRV and GRL142 were determined as previously described, with modifications^28,29^**.** The change of the fluorescence intensity associated with the protease -elicited cleavage of the fluorogenic substrate Arg-Glu(EDANS)-Ser-Gln-Asn-Tyr-Pro-Ile-Val-Gln-Lys(DABCYL)-Arg (HIV protease substrate 1; Sigma-Aldrich) was measured. All preparation procedures were done on ice, and the enzymatic reaction was processed at 37 °C. Enzymatic assays were performed in 0.1 M sodium acetate, 1.0 M sodium chloride, 1.0 mM ethylenediaminetetraacetic acid (EDTA), 1.0 mM dithiothreitol (DTT), and 1 mg/ml bovine serum albumin (BSA) pH 4.7 buffer containing various concentrations of a test compound with 30 µM substrate and 25 nM of protease. Fluorescence was measured using a Cytation 5 Cell Imaging Multimode Reader (BioTek, Winooski, VT) with an excitation wavelength of 340 nm and emission wavelength of 490 nm, and the relative fluorescent units (RFU) were obtained. Maximum velocity (V_max_) was calculated on Gen5 (BioTek), and the potency of HIV-1 protease activity inhibition by each compound was determined based on its reduced effectiveness on V_max_. Enzymatic assays for determination of the catalytic rate constant (k_cat_) and the Michaelis constant (K_m_) were also conducted under the same conditions. To determine the number of fluorophores out of RFU values, a standard curve was generated with tenfold serial dilutions of EDANS (AnaSpec, Fremont, CA), and the RFU values for each sample were converted to the numbers of EDANS molecules. IC_50_ values and Morrison K_i_ and K_m_ values were calculated on Prism 7 (GraphPad Software, La Jolla, CA).

## Crystallization of Darunavir-PR_p51_ and GRL142-PR_p51_ complexes

The unfolded PR_DRV_^R^_p51_ was refolded with the addition of a neutralizing buffer A (100 mM ammonium acetate pH 6.0, 0.005% Tween-20), making the final pH 5.0 to 5.2. The PR_DRV_^R^_p51_-containing solution was run through Amicon Ultra-15 10 K centrifugal filter units (Millipore), giving a solution containing PR (2 ~ 5 mg/ml) in 10 mM ammonium acetate pH 5.0 and 0.005% Tween-20. Occasionally, twice greater concentrations of DRV and GRL142 were used for crystallization. After centrifugation, the supernatants were collected and subjected to crystallization using the hanging-drop vapor diffusion method. Nextal Tubes ProComplex Suite (QIAGEN) was used for the first screening to determine the optimum crystallization condition. PR_DRV_^R^_p51_-DRV complexes were formed in 0.15 M (NH_4_)_2_SO_4_, 0.1 M HEPES pH 7.0, 20% (w/v) PEG 4000. PR_DRV_^R^_p51_-GRL142 complexes were formed in 0.15 M (NH_4_)_2_SO_4_, 0.1 M HEPES pH 6.8, 15% (w/v) PEG 4000. Crystals of PR_DRV_^R^_p51_ complexed with DRV or GRL142 were retrieved, immersed into cryoprotective solution containing reservoir solution plus 30% glycerol, and flash-frozen in liquid nitrogen.

**X-ray diffraction data collection.** The X-ray diffraction data of the PR_DRV_^R^_p51_ -DRV crystal were collected under cryogenic temperatures at SPring-8 BL44XU using X-rays of 0.9 Å wavelength and a CCD detector MX225-HE (Rayonix). A total of 180 diffraction images were collected using a rotation step of 1.0 degree with a camera distance of 161 mm. The diffraction data of the PR_DRV_^R^_p51_—GRL142 crystal were collected under cryogenic temperatures at SPring-8 BL41XU using X-rays of 1.0 Å and a pixel array detector PILATUS3 6 M detector (DECTRIS). A total of 360 images were collected using a rotation step of 1.0 degree with a camera distance of 210 mm.

**Structure solutions and refinement.** Solvent content and Matthew’s coefficient values were determined using CCP4 suite of programs^30,31^. GRL142 was fit using ARP/wARP – Ligands through CCP4^32,33^. The initial coordinates for GRL142 were taken from PDB ID 5TYS. Solvent molecules were fit using ARP/wARP-solvent through CCP4. The models were then refined using the simulated annealing protocol in phenix.refine through Phenix suite of programs^34^. Refinement libraries for GRL142 were optimized using the eLBOW-AM1 method and were manually checked through REEL as described previously^8,35^. For the darunavir–PR_p51_ complex, the structure 6OGL was used as the search model for molecular replacement using Molrep^36^. Darunavir (PDB ligand code: 017) was manually fitted into the structure. The model was refined using Refmac within the CCP4i2 interface. The 9CTX structure (PR_p51_ + darunavir) contains one molecule per asymmetric unit, whereas the PR_p51_ + GRL142 complex has two molecules in the asymmetric unit. MolProbity analysis showed no Ramachandran and rotamer outliers, with 98% of residues in favored regions for 9CTX; similarly, 6MKL exhibited no Ramachandran and rotamer outliers, with 99% of residues in favored regions. A summary of structure solution refinement statistics is given in supplementary Table 1.

**Protein structure accession IDs.** The final refined coordinates of PR_p51_-DRV and PR_p51_-GRL142 along with their corresponding X-ray data were deposited in RCSB Protein Data Bank (https://www.rcsb.org/) with accession IDs 9CTX and 6MKL, respectively.

**Molecular dynamics (MD) simulation.** The four crystal structures with PDB IDs 9CTX (PR_p51_-DRV), 6MKL (PR_p51_-GRL142), 4HLA (PR_wt_-DRV), and 5TYS (PR_wt_-GRL142) were used for determining the polar, nonpolar interactions and in carrying out MD simulations. Using the ‘protein preparation’ tool in Maestro, hydrogens were added, protonation states were assigned, and restrained minimization was carried out. OPLS3 force field was utilized in the simulations. A cutoff distance of 3.0 Å between a polar hydrogen and an oxygen or nitrogen atom, a minimum donor angle of 60° between D-H-A, and a minimum acceptor angle of 90° between H-A-B were used to define the presence of hydrogen bonds (“D,” “A,” and “B” stand for “donor,” “acceptor,” and “atom connected to acceptor,” respectively). Molecular surfaces for the inhibitors and selected PR residues from the active site were generated using a water sphere with a radius of 1.4 Å as a probe. Software tools from Schrödinger, LLC, New York, NY, as implemented in the Maestro version 10.7.015 (Schrödinger, LLC, New York), were used for model building, visualization, analysis and in generating the structural figures.

MD simulation was carried out on fully minimized and equilibrated structures using DESMOND^37^ as implemented within Maestro and briefly described as follows. For PR_p51_-DRV, the molecular system for carrying out molecular dynamics was built using the ‘System Builder’ option using the SPC solvent model (water as solvent) in an orthorhombic box, neutralized by adding three chloride ions, and adding 0.15 M NaCl. Similar protocols were utilized for preparing the PR_wt_-DRV, PR_wt_-GRL142, and PR_p51_-GRL142 complexes. The box volumes were minimized, and the systems were equilibrated. MD simulations were carried out for 1500 nano seconds, with timesteps of one femtosecond, and a electrostatic cutoff at 9 Å. The simulation trajectories were saved. The trajectories were analyzed at intervals of 0.5 ns to determine structural changes and fluctuations. The simulations were carried out using NVIDIA GPUs on the NIH Biowulf cluster.

The binding site volumes were calculated using the python script ‘trajectory_binding_site_volumes.py’ from Schrodinger, LLC. The script uses the SiteMap module to compute the binding site volume. Volumes for the PR_wt_ and PR_p51_ complexes from the crystal structures were computed. From the MD simulation trajectories, active site volumes at 0.5 ns intervals were computed.

## Supplementary Information

Supplementary Information.

## Data Availability

Data deposition, atomic coordinates and structure factors have been deposited in the Protein Data Bank, http://www.rcsb.org (PDB ID 9CTX and 6MKL). All other study data are included in the article and/or SI Appendix.

## Abbreviations

HIV_p51_: HIV_DRV_^R^_p51_
PR: Protease
PR_p51_: PR_DRV_^R^_p51_
PI: Protease inhibitor
DRV: Darunavir
Wt: Wild-type
vdW: Van der Waals
