# Supplementary material for: Distal amino-acid substitutions contribute to HIV protease inhibitor resistance as directly as proximal amino-acid substitutions
Source: Sci Rep. 2026 Oct 5;16:30880. doi: 10.1038/s41598-026-64620-7 (PMC13638461; doi:10.1038/s41598-026-64620-7)
Supplement: Supplementary file 1 [file 41598_2026_64620_MOESM1_ESM.pdf]

Supp. Fig. 1

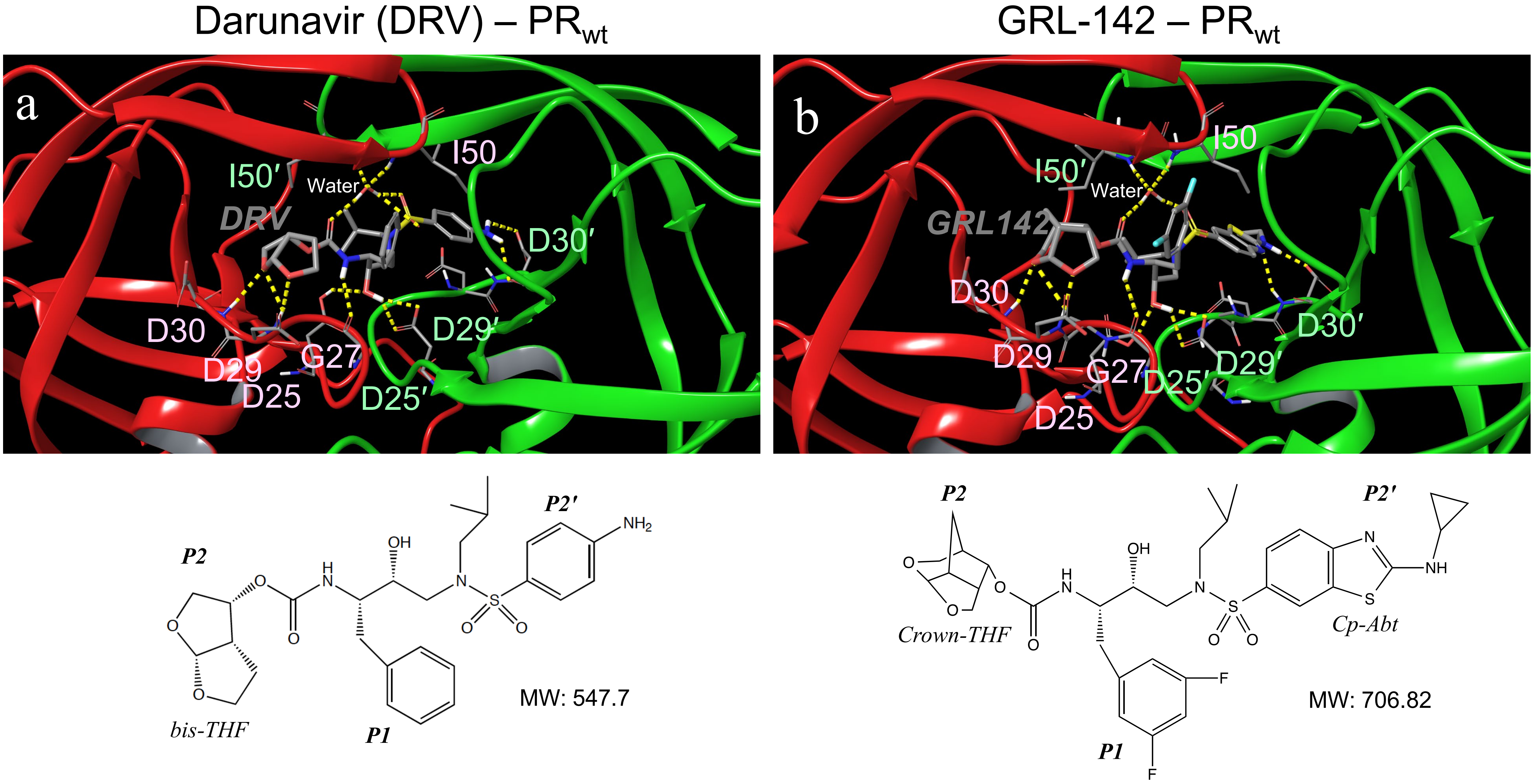
Darunavir (DRV) – PRwt
GRL-142 – PRwt
a
I50
I50′
Water
DRV
D30′
D30
D29′
D29
G27
D25
D25′
b
I50
I50′
Water
GRL142
D30
D30′
D29
D29′
G27
D25′
D25
P2
P2′
P1
bis-THF
NH2
OH
MW: 547.7
P2
P2′
P1
Crown-THF
Cp-Abt
NH
OH
F
F
MW: 706.82

Supp. Fig. 2

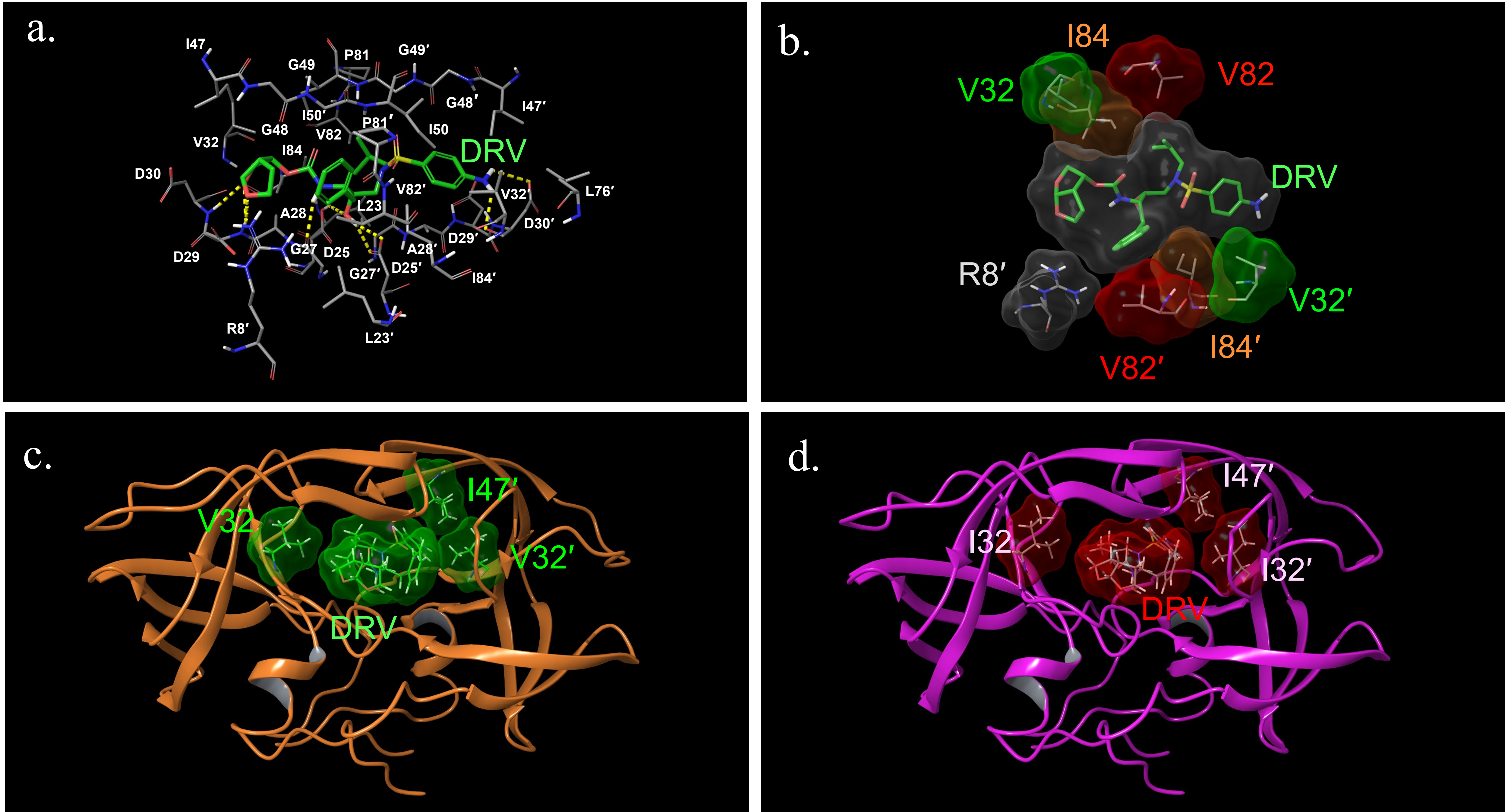

a.
I47
G49
P81
G49′
G48′
I47′
I50′
P81′
I50
G48
V82
V32
I84
DRV
D30
V82′
V32′
L76′
A28
L23
D30′
D29′
G27
D25
A28′
D29
G27′
D25′
I84′
R8′
L23′
b.
I84
V82
V32
DRV
R8′
V32′
I84′
V82′
c.
I47′
V32
V32′
DRV
d.
I47′
I32
I32′
DRV

Supp. Fig. 3

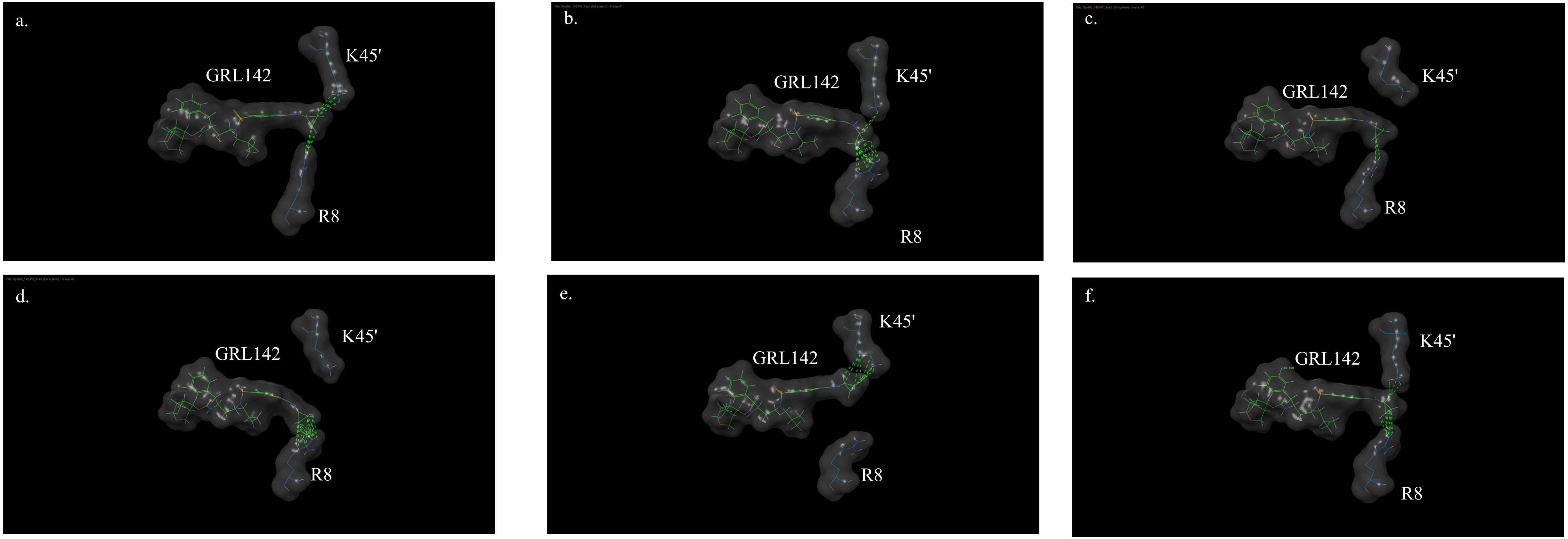
a.
GRL142
K45'
R8
b.
GRL142
K45'
R8
c.
GRL142
K45'
R8
d.
GRL142
K45'
R8
e.
GRL142
K45'
R8
f.
GRL142
K45'
R8

Supp. Fig. 4

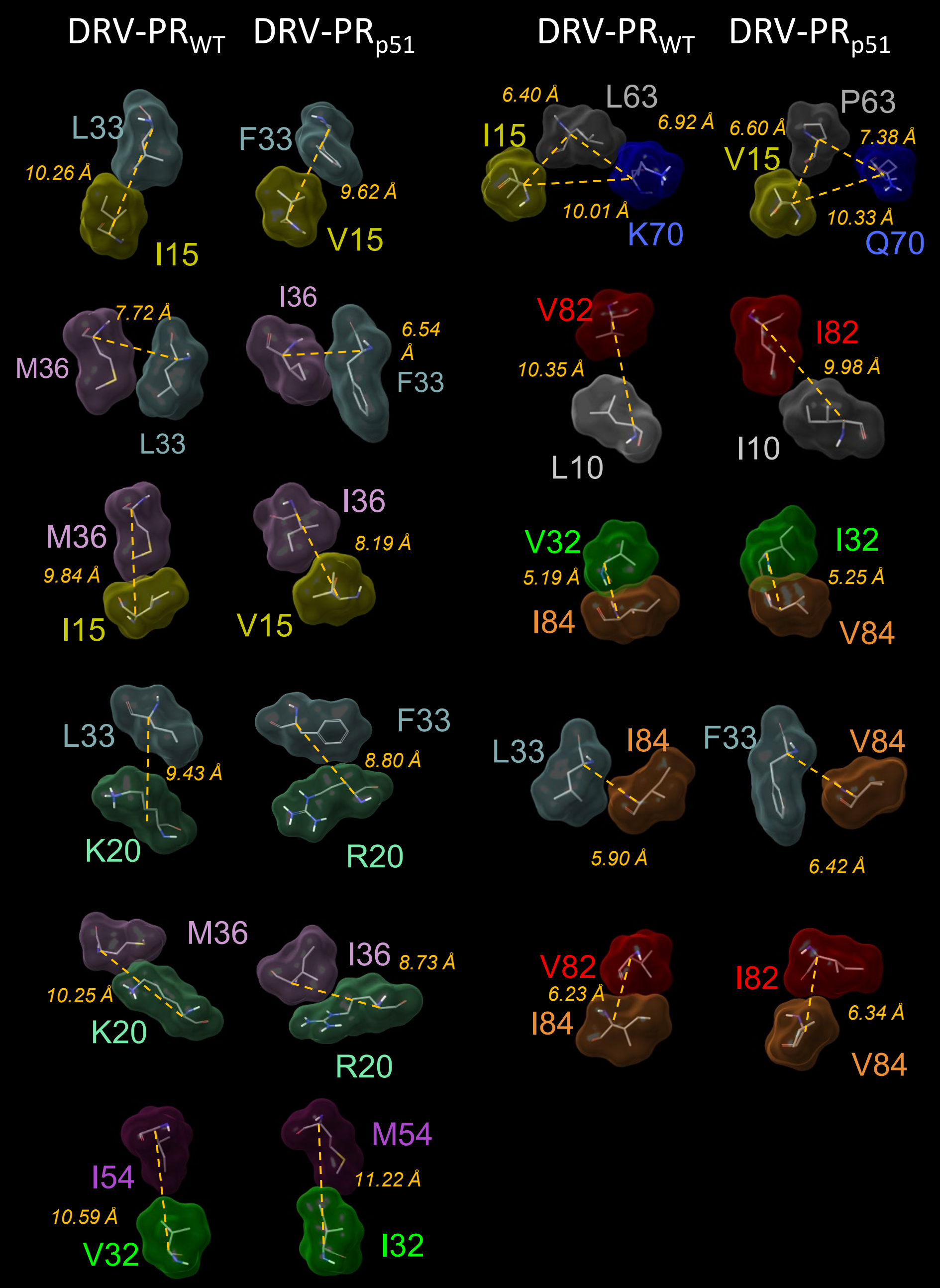

DRV-PRWT
DRV-PRp51
DRV-PRWT
DRV-PRp51
L33
10.26 Å
I15
F33
9.62 Å
V15
6.40 Å
L63
6.92 Å
I15
10.01 Å
K70
6.60 Å
P63
7.38 Å
V15
10.33 Å
Q70
7.72 Å
M36
L33
I36
6.54 Å
F33
V82
10.35 Å
L10
I82
9.98 Å
I10
M36
9.84 Å
I15
I36
8.19 Å
V15
V32
5.19 Å
I84
I32
5.25 Å
V84
L33
9.43 Å
K20
F33
8.80 Å
R20
L33
I84
5.90 Å
F33
V84
6.42 Å
M36
10.25 Å
K20
I36
8.73 Å
R20
V82
6.23 Å
I84
I82
6.34 Å
V84
I54
10.59 Å
V32
M54
11.22 Å
I32

Supp. Figure 5

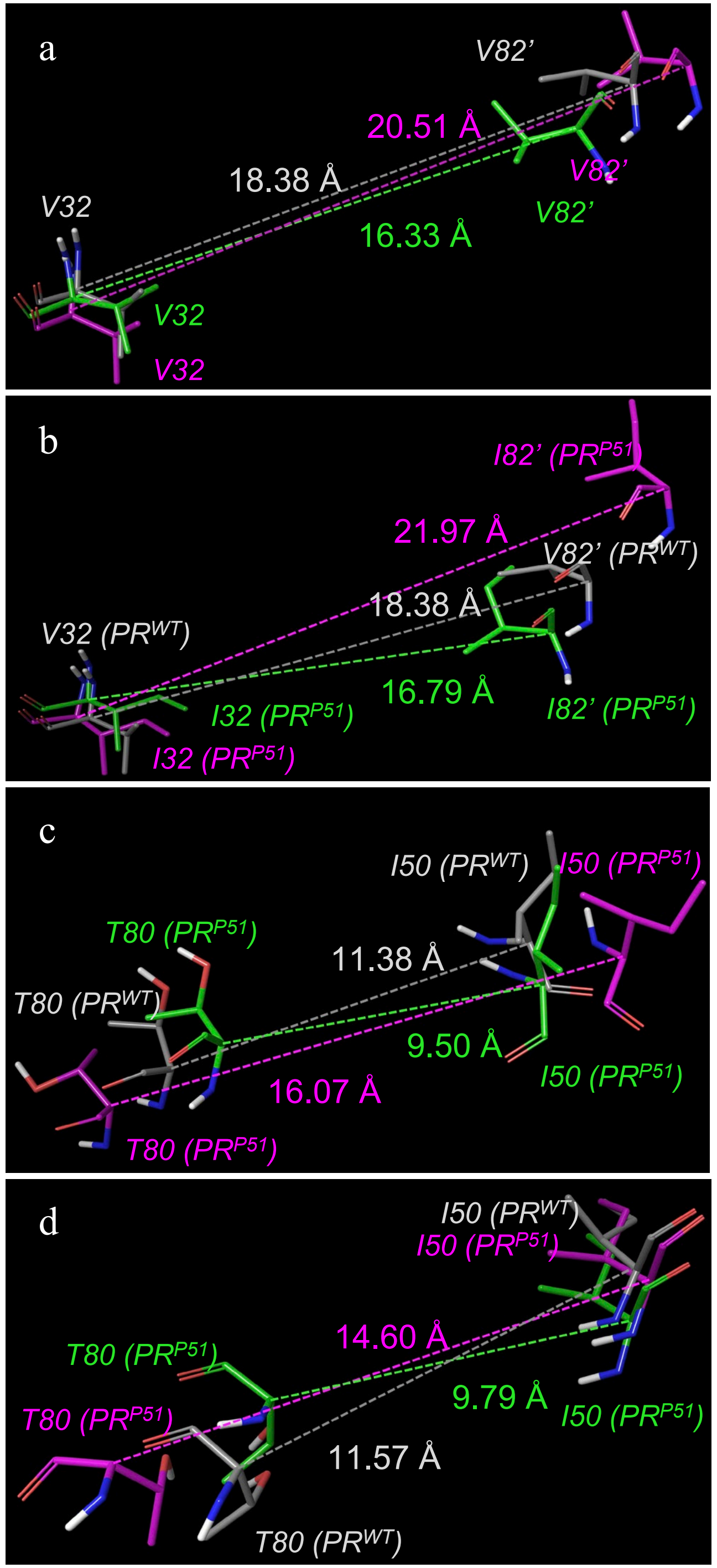

Supplementary Table 1. X-ray data collection and refinement statistics (molecular replacement)

Complex<br>PDB ID	PR_{p51} + DRV<br>9CTX	PR_{p51} + GRL142<br>6MKL
**Data collection**		
Space group	P 61 2 2	P 61
Cell dimensions		
a, *b*, *c* (Å)	62.68, 62.68, 81.76	63.28, 63.28, 82.07
α, β, γ (°)	90.00, 90.00, 120.00	90.00, 90.00, 120.00
Resolution (Å)	45.22 (1.55)*	50.0 (1.7)*
R_{sym} or R_{merge}	0.1525 (0.9386)	0.07 (0.56)
$I / \sigma I$	10.73 (0.92)	22.28 (2.3)
Completeness (%)	99.8 (91.7)	98.3 (97)
Redundancy	20.1 (13.5)	5.1 (5.2)
**Refinement**		
Resolution (Å)	45.22 (1.55)*	50.0 (1.7)*
No. reflections	14,085 (705)	39,949 (1,428)
R_{work} / R_{free}	0.165 / 0.208	0.168 / 0.188
No. atoms		
Protein	783	1528
Ligand/ion	85	96
Water	58	197
**B-factors**		
Protein	21.5	18.41
Ligand/ion	17.5	14.00
Water	29.4	30.44
R.m.s. deviations		
Bond lengths (Å)	0.0092	0.009
Bond angles (°)	2.158	1.288

*Values in parentheses are for the highest-resolution shell.

SUPPLEMENTARY FIGURE CAPTIONS

Supp. Fig 1. Analysis of crystal structure complexes show that DRV and GRL-142 have hydrogen bond interactions with the same PR_{wt} residues. The polar interactions of DRV and GRL142 with PR_{wt} are shown in panel a and panel b, respectively. The protease dimer is shown in red and green ribbons. DRV and GRL-142 are shown in thick sticks. Protease residues are shown in thin sticks. Hydrogen bonds are shown in yellow dotted lines.

Supp. Fig 2. There are subtle changes in the surface interactions of DRV with PR_{p51} compared to its interactions with PR_{wt}. **Panel a**: The PR_{wt} residues in the active site (<4 Å of DRV) of DRV-PR_{wt} complex (PDB ID: 4HLA) are shown: They are Arg8′, Leu23, Leu23′, Asp25, Asp25′, Gly27, Gly27′, Ala28, Ala28′, Asp29, Asp29′, Asp30, Asp30′, Val32, Val32′, Ile47, Ile47′, Gly48, Gly48′, Gly49, Gly49′, Ile50, Ile50′, Leu76′, Pro81, Pro81′, Val82, Val82′, Ile84 and Ile84′. **Panel b**: The surface interactions between DRV and selected active site residues in the DRV-PR_{wt} complex are shown. **Panels c-d**: The surface interactions of DRV with Val32, Val32′ and Ile47′ (green surfaces) of PR_{wt} (orange ribbons) have subtle differences from the surface interactions of DRV with Ile32, Ile32′ and Ile47′ (red surfaces) of PR_{p51} (purple ribbons).

Supp Fig. 3. Sample interactions between P2’ cyclopropyl group of GRL142 with Arg8 and Lys45’ of PR_{wt} from the MD simulation are shown in panels a-f. The vdW surfaces of GRL142, Arg8 and Lys45’ are shown. The green dashed lines show favorable interactions between atoms as determined by Maestro version 14.3. The analyses demonstrated that the P2’ cyclopropyl group of GRL142 fluctuates between Arg8 and Lys45’and interacts with them.

Supp Fig. 4. Substitutions from PR_{wt} to PR_{p51} results in a change in distance between the alpha carbons and in changes to the residue surface contact areas. The distance changes are large for L33/M36 (DRV-PR_{wt}) to F33/I36 (DRV-PR_{p51}); I15/M36 (DRV-PR_{wt}) to V15/I36 (DRV-PR_{p51}); K20/M36 (DRV-PR_{wt}) to R20/I36 (DRV-PR_{p51}). Changes in surface interactions are visually discernable for many residues.

Supp. Fig 5. The closest and farthest distances between residues show the range of distance fluctuation in MD simulation compared to the distance in the wild-type protease crystal structure. The distance between selected residues as observed in the DRV-PR_{wt} or GRL142-PR_{wt} crystal structure (gray fonts, gray carbons), the closest (green fonts, green carbons) and farthest (magenta fonts, magenta carbons) distances between these residues from MD simulation are shown. **Panel a** shows the distance between V32 and V82′ for DRV-PR_{wt}; **Panel b** between I32 and I82 for DRV-PR_{p51}; **Panel c** between I50 and T80 for DRV-PR_{p51}; and **Panel d** between I50-T80 for GRL142-PR_{p51}.
